# Mycoviruses diversity in the black kōji mold, *Aspergillus luchuensis* (section *Nigri*) isolated from liquor-production environments in Japan

**DOI:** 10.1016/j.virusres.2026.199724

**Published:** 2026-04-05

**Authors:** Hideki Kondo, Misaki Nanaji, Hitomi Sugahara, Miki Fujita, Ida Bagus Andika, Nobuhiro Suzuki, Fujimori Fumihiro

**Affiliations:** a Institute of Plant Science and Resources, Okayama University, Chuou 2-20-1, Kurashiki, Okayama, 710-0046, Japan; b Graduate School of Tokyo Kasei University, 1-18-1 Kaga, Itabashi, Tokyo, 173-8602, Japan; c Northwest A&F University, Yangling, China; d Graduate School of Agricultural Science, Tohoku University, Sendai, Japan

**Keywords:** *Aspergillus luchuensis*, Section Nigri, Mycovirus, RNA-seq, Virus population, Genome segment, Fermentation, Island

## Abstract

Some fungal species in the genus *Aspergillus* are economically important due to their role in the production of liquors and various foods; however, their viromes, which may affect their performance, remain unexplored. Therefore, this study examined the viromes of nine strains of *Aspergillus luchuensis* (section *Nigri*), the black kōji mold used in the production of shochu (a traditional Japanese liquor) in Japan. It identified virus-like sequences related to alterna-, partiti-, curvula, botourmia-, narna-like, and umbra-like viruses. Some sequences appear to represent new variants (e.g., alterna- and gammapartitiviruses), while many others correspond to novel viral species within established or proposed mycoviral families. All *A. luchuensis* strains harbored multiple virus infections, with 2 to 7 viruses per strain. Three alternavirus strains with four-segmented double-stranded RNA (dsRNA) genomes were confirmed, along with minor variants co-present with the predominant strains. Notably, a gammapartitivirus appears to have two additional dsRNA genome segments, along with two satellite-like short dsRNA segments in some fungal isolates. Furthermore, at least five short RNAs (0.48–1.31 kb) were identified, three of which are possibly satellite-like RNAs associated with novel single-stranded RNA viruses (botourmia- and umbra-like viruses). These findings reveal the great diversity of mycoviruses in *A. luchuensis* populations and lay the foundation for further investigation into their impact on fungal phenotypes and liquor production.

## Highlights

• Nine mycoviruses were identified from nine *Aspergillus luchuensis* strains.
• They are included alterna-, parti-, botourma-, narna- and umbra-like viruses.
• Five of the nine discovered mycoviruses represent novel viral species.
• *A. luchuensis* strains harbor 2 to 7 viruses.
• Additional genome segments and satellite-like RNAs are associated with four mycoviruses.

## Graphical abstract

**Figure:**
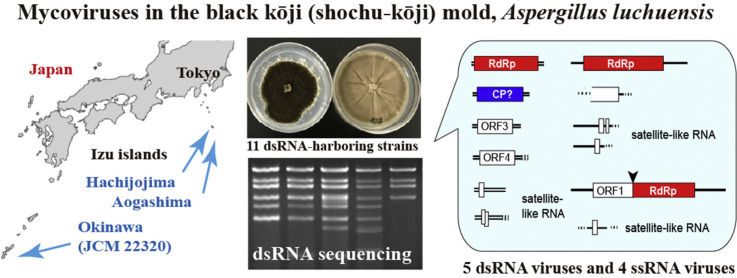

## 1 Introduction

The genus *Aspergillus* comprises over 340 officially recognized species of filamentous ascomycetous fungi that are widespread in the environment (Park et al., 2017). Some *Aspergillus* species, such as *A. fumigatus, A. flavus* and *A. niger,* act as opportunistic pathogens and allergens in humans, cause plant diseases, and produce mycotoxins, including aflatoxins. *Aspergillus* species are also important in industries, including food fermentation and the large-scale production of enzymes, organic acids, and pharmaceuticals (Navale et al., 2021; Park et al., 2017). Fungal species belonging to the two *Aspergillus* sections, *Nigri* and *Flavi. A. oryzae* and *A. sojae* (*Flavi*) and *A. luchuensis* (*Nigri*) have been used in food fermentation in Japan and other East Asian countries for a long time (Hong et al., 2013; Yamashita, 2021). The black kōji mold (*A. luchuensis*) and the white kōji mold (*A. luchuensis* mut. *kawachii*) are used to produce shochu and makgeolli (traditional distilled spirits and rice wine) as well as other foods and beverages (Futagami, 2022). As a member of section *Nigri, A. luchuensis* produces high levels of citric acid, which lowers the pH of fermentation mash, thereby preventing contamination by environmental microorganisms and enabling stable shochu production in relatively warm regions (Hong et al., 2013). Importantly, unlike their relatives (*A. flavus, A. parasiticus,* and *A. niger*), these fermentation-related *Aspergillus* species have lost their ability to produce mycotoxins such as aflatoxin, ochratoxin, and fumonisin (Tanaka et al., 2002; Yamada et al., 2016).

Mycoviruses are viruses that infect fungi and commonly persist in a latent state in their hosts (Ghabrial and Suzuki, 2009; Kondo et al., 2022). Several mycoviruses have been identified in the genus *Aspergillus*, particularly in species such as *A. fumigatus, A. flavus, A. niger,* and *A. foetidus*. These include double-stranded RNA (dsRNA) viruses, such as members of the *Partitiviridae, Chrysoviridae, Pseudototiviridae, Polymycoviridae* and *Alternaviridae*, as well as positive-sense, single-stranded RNA (+ssRNA) viruses, including members of *Botourmiaviridae, Narnaviridae, Mitoviridae* and *Yadokariviridae* families (Battersby et al., 2024; Degola et al., 2021; Fadli et al., 2025; Kotta-Loizou and Coutts, 2017; Zoll et al., 2018). While the broader ecological impact of *Aspergillus* mycoviruses remains unclear, some physiological effects have been reported. For instance, polymycoviral infection can induce hypovirulence (reduced virulence) or sensitize to a drug (Nikkomycin Z) and other stresses in *A. fumigatus* (Sass et al., 2023; Takahashi-Nakaguchi et al., 2020; Xie and Jiang, 2024), but in other cases it may enhance pathogenicity in a mammalian host (a model mouse) or in the insect *Galleria mellonella* (greater wax moth) (Kanhayuwa et al., 2015; Rocha et al., 2025). In *A. flavus*, mycoviral infection can affect aflatoxin production (Schmidt et al., 1986). However, information on mycoviruses in food fermentation-related *Aspergillus* species and their effects on fungal hosts remains very limited.

This study examined the viromes of nine *A. luchuensis* strains, identifying five dsRNA viruses (alterna-, partiti-, and curvulaviruses) and four +ssRNA viruses (botourmia-, narna-like, and umbra-like viruses), representing new strains or novel viral species. Multiple viral infections were observed across strains, ranging from two to seven mycoviruses per strain. Moreover, at least five short RNAs were identified, of which three were potentially associated with ssRNA viruses. These results provide further insights into the diversity of mycoviruses and their associated subviral RNAs in the genus *Aspergillus*.

## 2 Materials and methods

### 2.1 Fungal strains and culture conditions

**Fig. 1:**
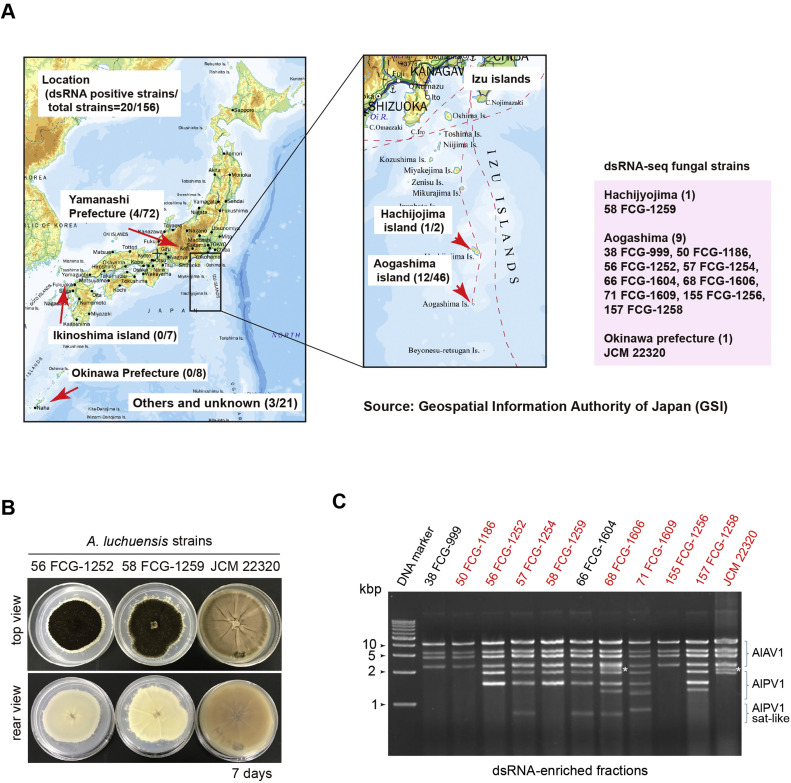
**Identification of mycoviruses in *A. luchuensis* strains.** (A) Origin of *Aspergillus* strains for dsRNA screening and the results of analysis. The number in parentheses indicates the number of dsRNA positive and tested *Aspergillus* spp. strains (Nanaji and Fujimori, unpublished data, see also the text). The map is provided by the Geospatial Information Authority of Japan (https://www.gsi.go.jp). The dsRNA-sequencing strains are shown on the right with an additional Okinawa strain (JCM22320, Table 1). (B) Colony morphologies of selected *A. luchuensis* strains carrying virus-like dsRNA elements. (C) dsRNA profiles of the selected *A. luchuensis* strains. Each dsRNA fraction was analyzed by 1.0% agarose gel electrophoresis. Fungal strains subjected to dsRNA-seq analysis are indicated in red. Expected dsRNA bands for an alternavirus (AlAV1) and a partitivirus (AlPV1) genome, as well as AlPV1 satellite-like elements, were shown on the right (see Table 2). The asterisks indicate the expected dsRNA bands for two other partitiviruses, AlPV2 and AlPV3. A 1 kb dsDNA ladder size marker (GeneRuler 1 kb DNA ladder, Thermo Fisher Scientific, Waltham, MA, USA) was used as a size standard.

The 12 dsRNA-positive strains of *A. luchuensis* were isolated from the following locations in Japan (Table 1 and Fig. 1A): Aogashima (nine strains) and Hachijojima (one strain) in the Izu Islands, and Okinawa Island (one strain: i.e., *A. awamori* var. *fuscus*). They were obtained from the Fujisankei Communications Group (FCG) Research Institute, Inc., and the Japan Collection of Microorganisms (JCM) at the RIKEN BioResource Research Center in Japan. The strains were primarily isolated from black kōji mold mash or the kōji room environment (air sampling) at shochu distilleries. They were cultivated on potato dextrose agar (PDA, Difco, Detroit, MI, USA) medium at 25 °C or room temperature.

**Table 1:** Japanese *Aspergillus luchuensis* strains and their dsRNA-seq results.

strain name	lab code	geographic origin in Japan	shochu-brewers*	Source	Collection year	dsRNA-sequencing (BioProject: PRJDB40404)⁎⁎		
						DRR Run files	total reads	detected viruses
FCG-9999	38	Aogashima-Isaland, Tokyo	B	air	2012	n. a.	n. a.	n. a.
FCG-1186	50	Aogashima-Isaland, Tokyo	n. p.	soil	2013	DRR909486	62,943,232	AlAV1, (AlPV1), AlBOV1, (AlNLV1)
FCG-1252	56	Aogashima-Isaland, Tokyo	C	air	2013	DRR909487	79,755,556	AlAV1, AlPV1, AlBOV1, AlNLV1
FCG-1254	57	Aogashima-Isaland, Tokyo	A	air	2013	DRR909492	50,021,458	AlAV1, AlPV1, (AlPV2), AlBOV1, AlNLV1, AlULV1
FCG-1259	58	Hachijojima-Island, Tokyo	n. p.	air	2013	DRR909488	75,713,148	AlAV1, AlPV1, AlBOV1, AlNLV1, AlULV1
FCG-1604	66	Aogashima-Isaland, Tokyo	A	koji	2013	n. a.	n. a.	n. a.
FCG-1606	68	Aogashima-Isaland, Tokyo	A	koji	2013	DRR909489	71,319,308	AlAV1, AlPV1, AlBV1, AlBOV1, AlNLV1, AlULV1
FCG-1609	71	Aogashima-Isaland, Tokyo	A	koji	2013	DRR909490	69,933,998	AlAV1, AlPV1, AlPV3, (AlBV1), AlBOV1, AlNLV1, AlULV1
FCG-1256	155	Aogashima-Isaland, Tokyo	A	air	2013	DRR909493	60,699,764	AlAV1, (AlPV1), AlBOV1, AlNLV1
FCG-1258	157	Aogashima-Isaland, Tokyo	B	air	2013	DRR909491	70,231,084	AlAV1, AlPV1, AlBOV1, AlSAV1
JCM 22320	n. p.	Okinawa	n. p.	n. p.	1936	DRR909494	65,724,800	AlAV1, AlPV2
(IAM 2351)								

⁎ A–C represent three distinct anonymous shochu distilleries. n. p., not provided (unknown).

⁎⁎ see Table 2 for the virus abbreviations. Viruses with fewer NGS reads are indicated in parentheses (see Fig. 6); n. a., Not applicable.

### 2.2 RNA preparation and analyses

Total dsRNA was extracted from mycelia using a phenol:chloroform:isoamyl alcohol mixture (25:24:1; v/v) and then purified using B3 cellulose powder (Advantec Co., Ltd., Tokyo, Japan) (Urzo et al., 2024). Then, the dsRNA fractions were treated with RQ1 RNase-free DNase I (Promega, Madison, WI, USA) and S1 nuclease (Takara Bio Inc., Otsu, Japan). Total RNA was extracted from mycelia using either the conventional phenol/chloroform method or TaKaRa RNAisoPlus Reagent (TaKaRa Biotech. Co., Shiga, Japan). Then, the total RNA fractions were examined using 1% agarose gel electrophoresis in 1 × Tris-acetate-EDTA buffer (pH 7.8).

### 2.3 RNA-seq analysis

Total dsRNA samples from nine *A. luchuensis* strains (Table 1) were individually analyzed by next-generation RNA sequencing (RNA-seq) as described previously (Kondo et al., 2016). The complementary DNA (cDNA) libraries were constructed from heat-denatured dsRNAs using the TruSeq RNA Sample Preparation Kit v2 (Illumina, San Diego, CA, USA). Then the cDNA libraries were subjected to paired-end sequencing with a 100-nucleotide read length using the Illumina NovaSeq 6000 platform, performed by Macrogen Inc. (Tokyo, Japan). After sequencing, the adapter-trimmed raw sequence reads were assembled *de novo* using the CLC Genomics Workbench (version 20; CLC Bio-Qiagen, Aarhus, Denmark). Next the assembled contigs were subjected to local Blast searches against the viral reference sequence (RefSeq) dataset of the National Center for Biotechnology Information (NCBI), as described previously (Kondo et al., 2025). Then, the reads were mapped to each virus-like contig using the Read Mapping algorithm in CLC Genomics Workbench with a length fraction of 0.9 and a similarity fraction of 0.98.

### 2.4 Sequence analysis and database searching

To verify viral infection, reverse transcription–polymerase chain reaction (RT-PCR) assays were performed using specific primer sets for each virus candidate and the total RNA fraction from the *A. luchuensis* strains. To obtain the complete genomic or segment sequences, 3′-RNA ligase-mediated rapid amplification of cDNA ends (RLM-RACE) was performed using the dimethylsulfoxide (DMSO)/heat-denaturing dsRNA fractions as described previously (Lin et al., 2012). To verify a possible circular or concatenated nature of the virus genome, inverse RT-PCR assay was performed using a primer pair designed to span the 5′ and 3′ junction of the target genomic segment using denatured dsRNA fractions (Shamsi et al., 2022). Then, the PCR fragments were separated by electrophoresis in a 1% TAE agarose gel and then sequenced using the Sanger method. The primer information used in this study is available upon request. Virus or virus-like sequence data were analyzed using GENETYX-MAC version 22 (Genetyx Co., Tokyo, Japan) or Enzyme X (version 3.3.3; Nucleobytes B.V., Aalsmeer, The Netherlands). The electropherograms of sequence were shown using 4Peaks (version 1.8, Nucleobytes B.V.) software. Blast or reverse Blast searches were performed using NCBI’s non-redundant (nr) DNA and protein databases. The pairwise identity matrix for the viral genome comparison was calculated using Clustal Omega via the EMBL-EBI web server (https://www.ebi.ac.uk/jdispatcher/msa/clustalo).

### 2.5 Phylogenetic analysis

The identified mycoviruses were phylogenetically analyzed using a previously described method (Kondo et al., 2023) with slight modifications. First, the RNA-dependent RNA polymerases (RdRPs) were aligned with MAFFT (version 7) (Katoh and Standley, 2013) and trimmed with trimAI (version 1.41) to remove poorly aligned regions (Talavera and Castresana, 2007) on the ngphylogeny.fr web server (https://ngphylogeny.fr/tools/). Next, maximum likelihood (ML) trees were constructed using IQ-TREE with Shimodaira–Hasegawa approximate likelihood ratio test (SH-aLRT)/ultrafast bootstrap support values (Trifinopoulos et al., 2016). The best-fit model was selected using ModelFinder (Kalyaanamoorthy et al., 2017) on the IQ-TREE web server (http://iqtree.cibiv.univie.ac.at/). The tree topology was visualized using FigTree (1.4.4 software; https://tree.bio.ed.ac.uk/software/figtree/).

## 3 Results

### 3.1 *dsRNA detection on* Aspergillus *strains*

In total,156 *Aspergillus* strains obtained from the FCG Research Institute were screened for mycoviruses using the dsRNA extraction method (see Fig. 1A, Nanaji and Fujimori, unpublished data). These strains belong to *A. niger* and other species within the section *Nigri* species, including *A. tubingensis, A. japonicus* and *A. luchuensi.* They were mainly collected from wineries and vineyards in Yamanashi Prefecture as well as the shochu distilleries on the Izu Islands (Tokyo), Ikinoshima island (Nagasaki Prefecture), and Okinawa Prefecture (Table 1 and Fig. 1A). Of these strains, 20 (12.8%) were found to carry double-stranded RNA (dsRNA): 7 *A. niger,* 2 *A. japonicus* and 10 *A. luchuensis* strains. The first two species were primarily isolated from wineries and other locations, while *A. luchuensis,* also known as the black koji mold, was isolated from shochu distilleries (Fig. 1A and Table 1).

Nine of the 10 dsRNA-harboring *A. luchuensis* strain were isolated from Aogashima Island (38 FCG-999, 50 FCG-1186, 56 FCG-1252, 57 FCG-1254, 66 FCG-1604, 68 FCG-1606, 71 FCG-1609, 155 FCG-1256, and 157 FCG-1258), and one was isolated from Hachijojima Island (58-FCG-1259), both located in the Izu Islands (Table 1 and Fig. 1A). An additional dsRNA-harboring strain of *A. luchuensis* (JCM 22320) obtained from Okinawa Island was also included in the analysis (Sakaguchi, 1946). All 10 isolated dsRNA-harboring *A. luchuensis* strains exhibited dark-colored colonies, whereas JCM 22320 showed brownish-black colonies (Fig. 1B). All strains showed dsRNA profiles with 4–8 bands (Fig. 1C). Four similar dsRNA bands with sizes large than 2 kbp were present in all strains, while the presence and pattern of smaller dsRNA bands (<2 kbp) varied among strains.

### 3.2 Viromic analysis of the A. luchuensis strains

**Fig. 2:**
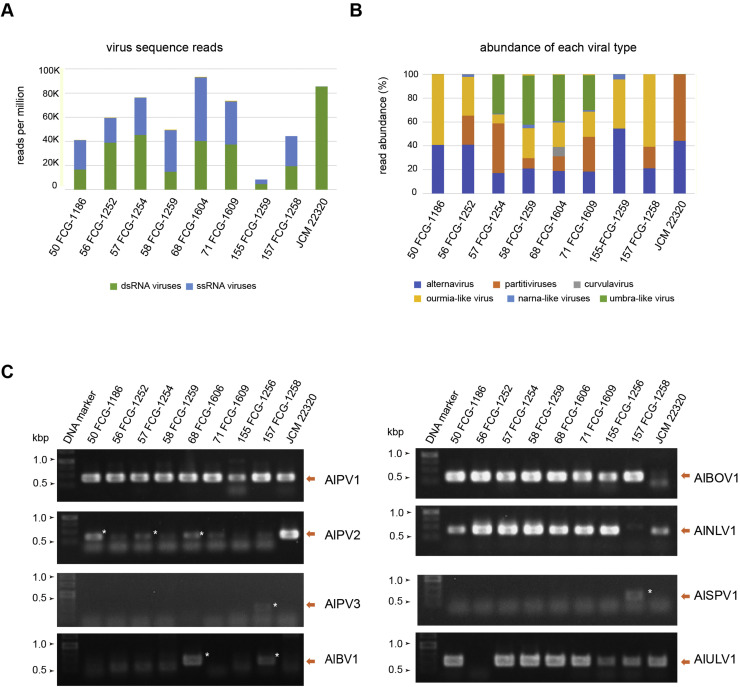
dsRNA sequencing and validation of virus presence. (A) Sum of the total reads of dsRNA or ssRNA viruses (normalized to reads per million) obtained from the RNA-seq analyses in each *A. luchuensis* strain. (B) Proportions of virus-related reads of each viral group in each *A. luchuensis* strain. (C) Validation of the presence of each candidate virus or viral segments by RT-PCR using total dsRNA from the original fungal strains (see Table 2 for virus abbreviations). Primers were designed to target the RdRP sequence of each candidate virus. An asterisk indicates a weak amplicon band derived from a virus.

Next, dsRNA fractions extracted from the above-described *A. luchuensis* strains were subjected to RNA-seq. Local Blast analysis of de novo-assembled contigs identified at least 30 unique virus-like sequences ranging from 1.1 to 3.6 kb that exhibited sequence similarity to known viruses or virus-like sequences (Table 2 and S1). The number of virus-derived reads ranged from approximately 2.6 to 6.2 millions in all strains except for 155 FCG-1259 (0.5 million). The number of +ssRNA virus reads was approximately 35–70% that of virus reads in all strains except JCM 22320, which lacked detectable ssRNA viruses (Fig. 2A). From these analyses, we identified five dsRNA viruses (an alternavirus, three partitiviruses, and a curvulavirus) and four +ssRNA viruses (a botourmiavirus, two narna-like viruses, and an umbra-like virus) with nearly complete genome sequences determined (Table 2). No negative-sense, ambisense RNA, or circular single-stranded DNA viruses were detected. Each *A. luchuensis* strain harbored multiple viruses belonging to at least two different families/groups, with alternaviruses and botourmia-like viruses distributed across nearly all examined strains (Fig. 2B).

**Table 2:** Viruses from Japanese *A. luchuensis* strains.

virus name	abbreviation	genus or group	species*	segments/variant	satellite	Accession
dsRNA viruses						
Aspergillus luchuensis alternavirus 1	AlAV1	*Alternavirus*	*Alternavirus aspergilli*	4/A	-	LC923879-LC923882
				4/B (B1)	-	LC923883-LC923886
				4/ C	-	LC923887-LC923890
Aspergillus luchuensis partitivirus 1	AlPV1	*Gammapartitivirus*	unassinged species (AfPV1)	2–4	2	LC923891-LC923897
Aspergillus luchuensis partitivirus 2	AlPV2	epsironpartitivirus	novel species	2	-	LC923898-LC923899
Aspergillus luchuensis partitivirus 3	AlPV3	*Betapartitivirus*	novel species	2	-	LC923900-LC923901
Aspergillus luchuensis bipartite virus 1	AlBV1	*Orthocurvulavirus*	novel species	2	-	LC923902-LC923903
ssRNA viruses						
Aspergillus luchuensis botourmiavirus 1	AlBOV1	*Magoulivirus*	novel species	1 (+1?)	2	LC923904-LC923907
Aspergillus luchuensis narna-like virus 1	AlNLV1	narna-like virus	novel species	2	-	LC923908-LC923909
Aspergillus luchuensis splipalmivirus 1	AlSPV1	*Divipalmivirus*	unassinged species (AfNV1)	3	-	LC923910-LC923912
Aspergillus luchuensis umbra-like virus 1	AlULV1	mycotombusvirus	unassinged species (EnULV2)	1	1	LC923913-LC923914

⁎ The bracketed viruses are considered to belong to the same unassinged species based on the 70% RdRP amino acid sequence identity criterion set in this study. For virus abbreviations, refer to the text.

**Fig. 3:**
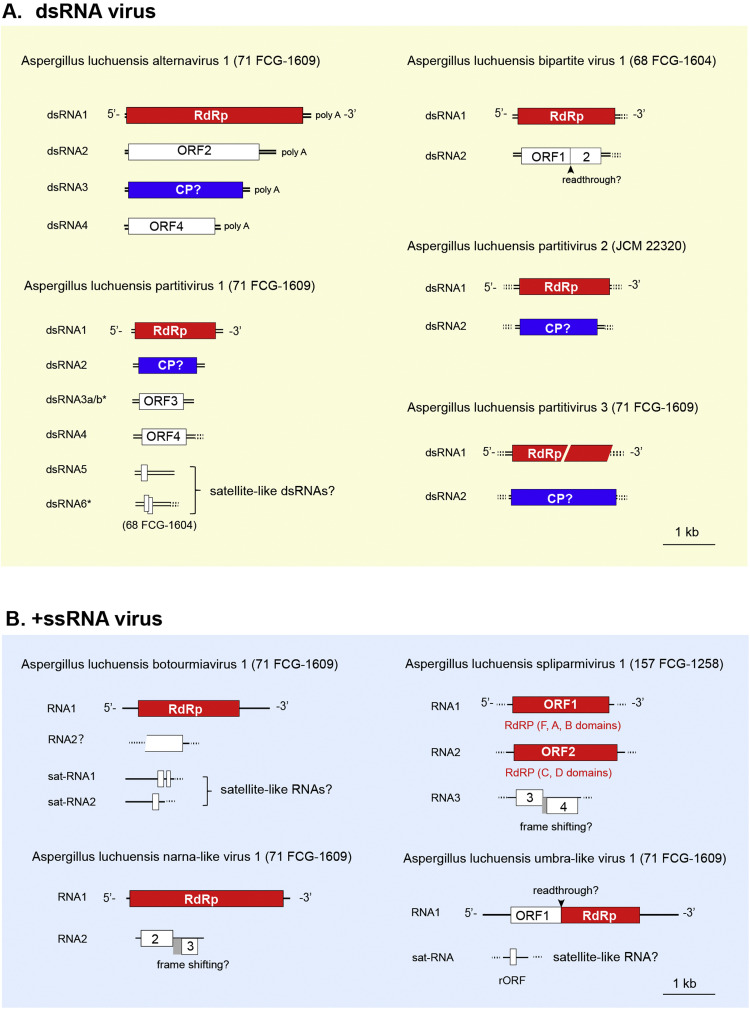
**Genome organization of the identified *A. luchuensis* viruses**. Schematic representation of dsRNA viruses (A): Aspergillus luchuensis alternavirus 1 (AlAV1) variant A, Aspergillus luchuensis partitivirus 1–3 (AlPV1–3) and Aspergillus luchuensis bipartite virus 1 (AlBV1) and ssRNA viruses (B): Aspergillus luchuensis botourmiavirus 1 (AlBOV1), Aspergillus luchuensis narna-like virus 1 (AlNLV1), Aspergillus luchuensis splipalmivirus 1 (AlSPV1) and Aspergillus luchuensis umbra-like virus 1 (AlULV1). The ORFs and segmented genome structures are indicated. The undetermined terminal sequence of certain virus segements is indicated by dotted lines, and predicted frameshifts or read-throughs are indicated as well. rORF is located on the reverse strand.

To further validate the presence of viruses in the *A. luchuensis* strains, RT-PCR targeting the RdRP sequences was performed (Fig. 2C and data not shown). The RT-PCR results were generally consistent with the RNA-seq results, and signal intensity correlated with read counts. In some instances, viruses undetectable by RNA-seq were detected by RT-PCR (e.g., the partitiviruses and umbra-like virus), possibly due to differences in the *A. luchuensis* cultures or strain batch used for RNA extraction. Complete or nearly complete genomic sequences of the several mycovirus isolates (alternavirus, partitiviruses, botourmiavirus, narna-like viruses, and umbra-like virus) infecting strains 71 FCG-1609 and 68 FCG-1606 were obtained using RLM-RACE analysis (Figs. 3 and S1 and Table S1).

### 3.3 Identified dsRNA viruses

**Fig. 4:**
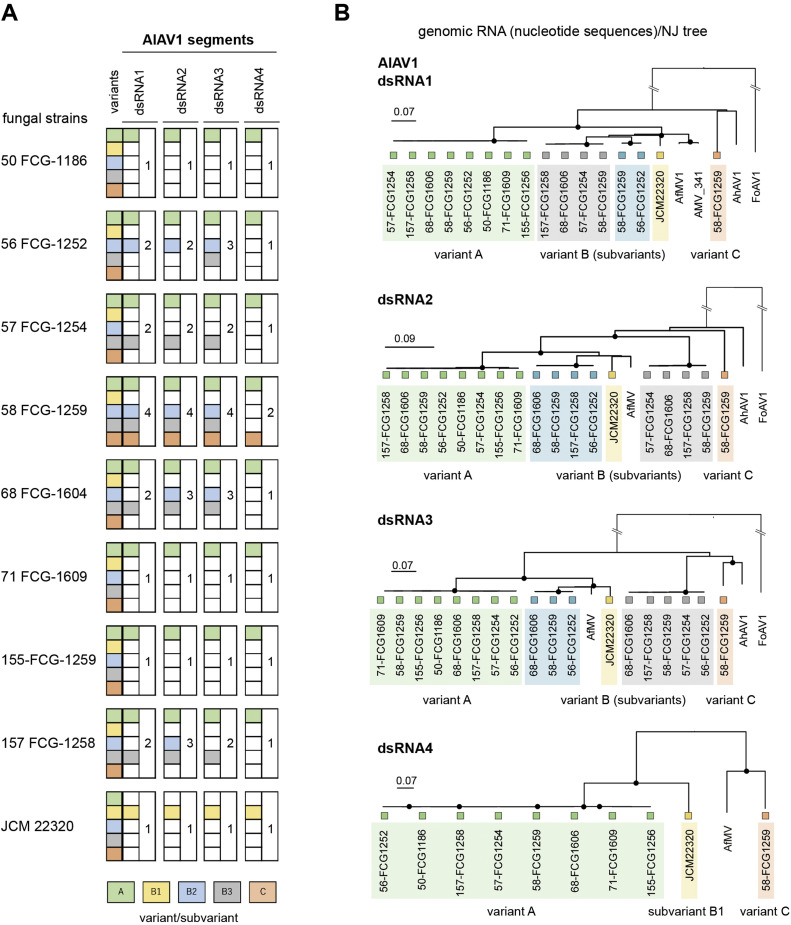
**Analysis of AlAV1 variants in *A. luchuensis* strains.** (A) The presence of each AlAV1 segment for variants A, B and C, including putative subvariants B1–B3. Each variant/subvariant is color-coded, and, the number (1–4) of variants identified in each strain per segment is shown. Representative AlAV1 sequences for each variant (A, B1, and C) have been deposited in public databases (see Tables 2 and S1). (B) Phylogenetic relationships of the AlAV1 variants/subvariants based on nucleotide sequences (NJ method). AfRV, Aspergillus foetidus dsRNA mycovirus, HE588144–HE588146, and HE647818; AMV_341, Aspergillus mycovirus 341, EU289897; AhAV1. Aspergillus heteromorphus alternavirus 1, MK279437–MK279439.

An alternavirus (family *Alternaviridae,* order *Ghabrivirales*) was identified from the *A. luchuensis* strains and named Aspergillus luchuensis alternavirus 1 (AlAV1) (Tables 2 and S1). Four high-molecular-mass dsRNA bands (>2 kbp) appear to correspond to its quadripartite genome segments (Figs. 1C and 3). AlAV1 has three major variants (A, B and C) based on the phylogenetic topology of AlAV1 dsRNA1 sequences (Fig. 4). The complete genomic sequences of the representative AlAV1 variant A, the most common variant infecting the strain 71 FCG-1609, were obtained (Table S1). The 5′ and 3′ terminal sequences were moderately conserved as previously reported for the Aspergillus foetidus dsRNA mycovirus (AfRV, also known as Aspergillus foetidus fast virus) (Kozlakidis et al., 2013c) (Fig. S1A). The proteins of all three major AlAV1 variants show high amino acid identity with AfRV proteins (92.9%–98.5%), whereas dsRNA4 proteins exhibit lower identity (45.2%–77.4%) (Table S1, and see also the RdRP ML tree in Fig. S2). The absence of dsRNA4 in AlAV1 minor variants and AfRV, coupled with the unknown function of this segment, suggests that it may not be essential for viral replication and may therefore be eliminated or left out during virus evolution. The significant discrepancy in sequence conservation also implies that dsRNA4 may have evolved distinct functional characteristics among different virus species. Blast analysis suggested that AlAV1 and AfRV belong to the same established species *Alternavirus aspergilli.* AlAV1 shares key features with the members of the genus *Alternavirus*, including a 3′ poly-A sequence on the positive-strand RNA and the ADD triplet within the RdRP, instead of the GDD, SDD, or GDN residues, that are common hallmarks of viral RdRP (te Velthuis, 2014). However, it remains to be elucidated whether the capping status of the 5′ end and the length of the 3′ poly A tail of genome segments of AlAV1 and other members share characteristics similar to those of Alternaria alternata virus 1 (Wu et al., 2021). AlAV1 variant B comprises of three sub-variants (B1, B2, and B3): B1 was obtained from the Okinawa strain JCM 22320, while B2 and B3 were obtained from the Aogashima and Hachijojima strains, respectively, and likely lack one or more segments. The actual combination of segments warrants further investigation.The B2 and B3 sub-variants were always found as mixed infections with the predominant variant (Fig. 4A). The AlAV1 variant C was detected in the Hachijojima Island strain 58 FCG-1259, which was coinfected with other AlAV1 variants.

**Fig. 5:**
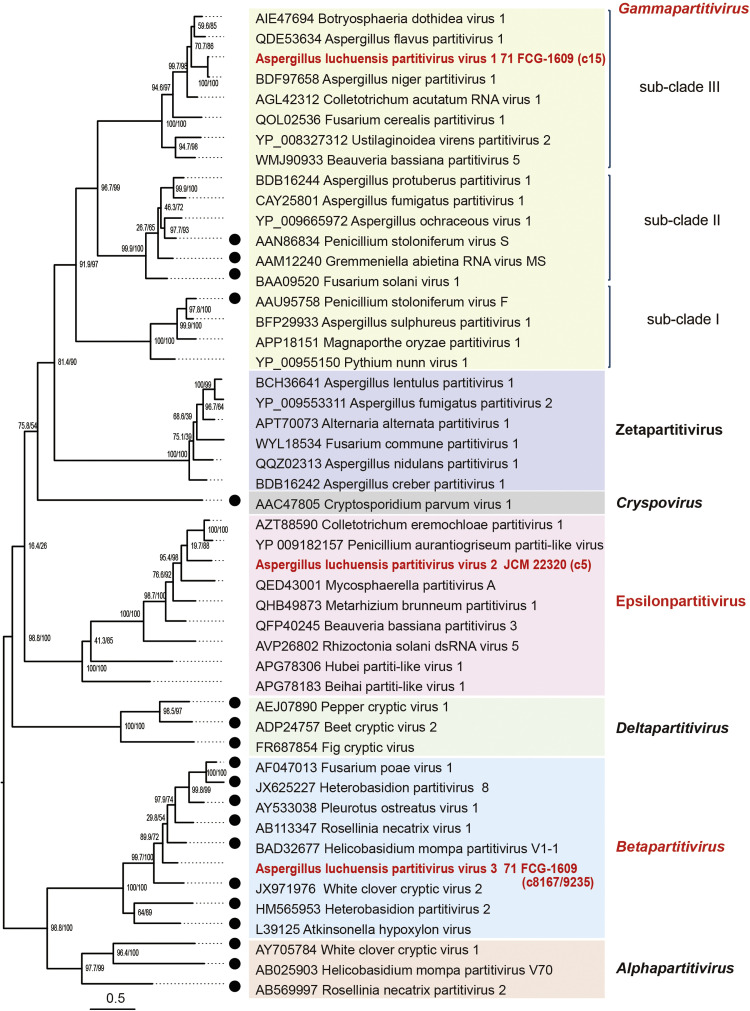
**Phylogenetic relationships of *A. luchuensis* partitiviruses**. A mid-pointed maximum likelihood (ML) tree was constructed based on the RdRP alignment of selected partitiviruses (family *Partitiviridae*, order *Durnavirales*) using the LG+F + I + G4 substitution model. Established viral species are indicated by filled circles, and viruses identified in this study (Aspergillus luchuensis partitivirus 1–3 [AlPV1–3]) are shown in bold with red letters (Tables 2 and S1). Clades are color-coded by genus or related groups. Branch numbers indicate SH-aLRT (%) / ultrafast bootstrap (%) support. The scale bar represents the number ofamino acid substitutions per site.

Several virus-like sequences related to partitiviruses (family *Patitiviridae,* order *Durnavirales*) were identified. Three were found in strains 71 FCG-1609 and JCM 22,320 and named Aspergillus luchuensis partitivirus 1 and 3 (AlPV1 and AlPV3) and 2 (AlPV2), respectively (Tables 2, S1 and Fig. 3A). Two additional virus-like sequences related to orthocurvulaviruses (family *Curvulaviridae,* order *Durnavirales*) were also detected in strain 68 FCG-1604 and named Aspergillus luchuensis bipartite virus 1 (AlBV1) (Table S1 and Fig. 3A). AlPV1 RdRP (dsRNA1) and capsid protein (CP, dsRNA2) exhibited 96.3 and 94.3% sequence identity, respectively, with the corresponding sequences of Aspergillus niger partitivirus 1 (AnPV1, a gammapartitivirus), suggesting that they belong to the same unassigned viral species (Table S1). Pairwise analysis of AlPV1 genomes showed over 98% nucleotide sequence identity between the respective contigs of dsRNA1 and dsRNA2. The full-length genome sequences of AlPV2 and AlPV3 have not yet been completely obtained, although terminal sequence alignment provides near-complete coverage (Fig. S1B). Blast analysis of RdRP identified Colletotrichum eremochloae partitivirus 1 (proposed genus “Epsilonpartitivirus”) for AlPV2 and Helicobasidium mompa partitivirus V1–1 (genus *Betapartitivirus*) for AlPV3, with 63% amino acid identity, as their closet relatives. According to current species demarcation criteria of the International Committee on Taxonomy of Viruses (ICTV, <90% identity for RdRP and <80% for CP), both viruses represent novel species (Vainio et al., 2018) (see Table S1 for CP genes). Phylogenetic analysis of RdRPs supports these taxonomic placements (Fig. 5).

**Fig. 6:**
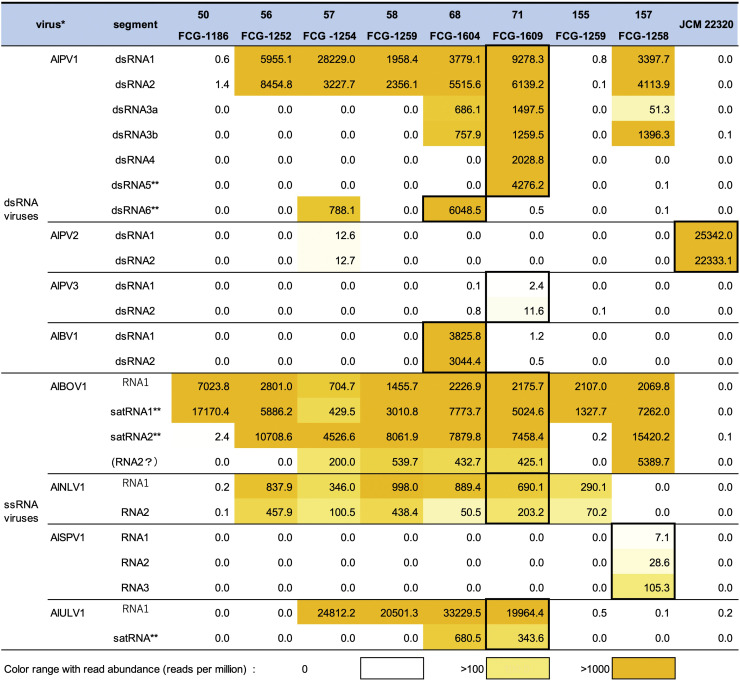
**Abundance of viral reads for each *A. luchuensis* dsRNA sample.** Read counts were determined using the reference sequences for dsRNA or ssRNA viruses listed in Tables 2 and S1. Potential viral segments or subviral RNAs are included. Counts were normalized to reads per million and displayed as a heat map. AlAV1 reads was not included. Refer to Fig. 4 for sequence information. *: reference contig sequences derived from each fungal strain were listed in Table S1 (thick-black-lined boxes); **: putative staellite-like elements.

**Fig. 7:**
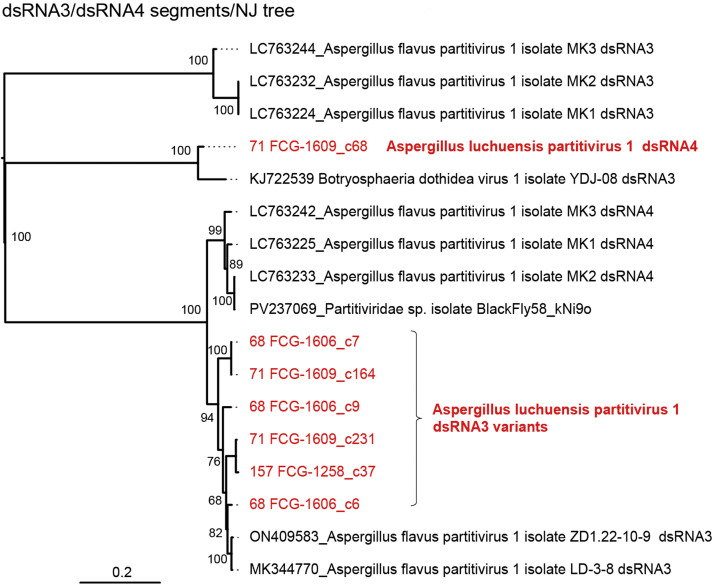
**NJ tree showing the phylogenetic relationships based on the nucleotide sequences of the AlPV1 dsRNA3 and dsRNA4.** Note that the annotation for dsRNA3 and dsRNA4 in AfPV1 varies depending on the isolates.

Some AlPV1 isolates (57 FCG-1654, 68 FCG-1604, 71 FCG-1609 and 157 FCG-1258) harbored smaller dsRNA segments in addition to the two primary segments (Table 2 and Fig. 6). For example, two variants of dsRNA3 (dsRNA3a and 3b) and dsRNA4 segments were present in strain 71 FCG-1609. The dsRNA3-encoded hypothetical protein shares 93.2% similarity with that of Aspergillus flavus partitivirus 1 (AfPV1) (Jiang et al., 2019), and the dsRNA4 protein exhibits 88.3% sequence identity with the dsRNA3-encoded protein of Botryosphaeria dothidea virus 1 (BdV1). Terminal sequences are partially conserved (Fig. S1B). They share conserved nucleotides at the 5′-KWACUUUUR…(ORF)…CCUYRAYYCA-3′ termini. Two additional small contigs (dsRNA5 and dsRNA6) from strains 71-FCG1609 and 68-FCG1604 strains were also confirmed to share terminal sequences partially with other segments and with each other (Fig. S1B). This suggests that they may represent non-coding satellite-like elements, as reported for AfPV1 (Jiang et al., 2022). However, given the presence of short ORFs (<100 amino acids) in dsRNA5 and dsRNA6, the possibility of their expression cannot also be ruled out (Wang et al., 2014) (Fig. 3, Fig. 6). AlPV1 was detected in at least six *A. luchuensis* strains. dsRNA3 was found in strains 68 FCG-1604, 157 FCG-1258, and 71 FCG-1609 (Fig. 6). The dsRNA3a and 3b variants were highly conserved between strains 71 FCG-1609 (c231) and 157 FCG-1258 (c37), and between strains 71 FCG-1609 (c164) and 68 FCG-1604 (c7), respectively (see neighbor-joining [NJ] tree in Fig. 7). Other two dsRNA3 variants (c6 and c9) in strain 68 FCG-1604 exhibited 3%–6% amino acid sequence divergence. dsRNA4 and dsRNA5 were detected only in strain 71 FCG-1609, whereas dsRNA6 was found in strains 57 FCG-1254 and 68 FCG-1604 (Fig. 6). Smaller dsRNA bands (<2 kbp) in agarose gels (Fig. 1C) likely correspond to these partitiviral dsRNA elements and associated satellite-like RNAs, as supported by the RNA-seq data (Fig. 6). Determining the relationships between these dsRNA elements and AlPV1, as well as their respective functions, represents an interesting objective for future research.

**Fig. 8:**
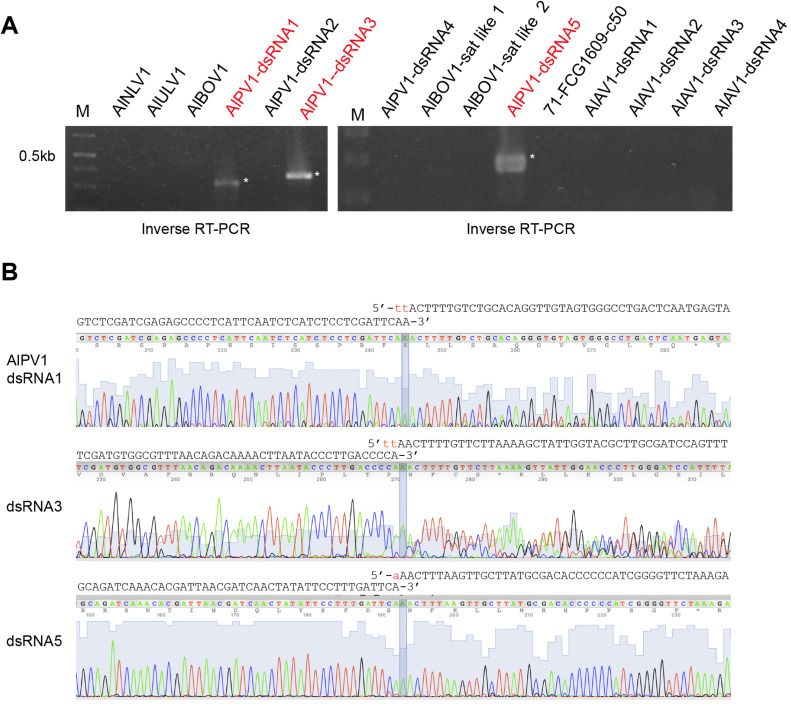
**Inverse RT-PCR analyses of the viral genome termini.** (A) Inverse RT-PCR using a primer set for AfPV1 and some other viruses. DMSO/heat denaturing dsRNA samples from strain 71-FCG1609 were used as templates for reverse transcription. An uncharacterized contig (71-FCG1609-c50) is also included. GeneRuler 1 kb DNA ladder was used as a size standard. (B) Sanger sequencing chromatogram of the inverse RT-PCR products. The resulting sequence trace files are shown using 4Peaks. A potential 3′–5′ junction sequence is highlighted in the chromatogram. The 3′ and 5′ terminal AlPV1 segments are indicated above each chromatogram. Red letters may indicate missing sequences in the inverse RT-PCR products.

Analysis of the AlPV1 contig sequences suggested that the 3′ and 5′ genomic ends may be concatenated head-to-tail in certain contigs (data not shown), reminiscent of the circular forms or oligomerzation of the viral genomes reported for +ssRNA mitoviruses (Shamsi et al., 2022). Consequently, inverse PCR was performed using the same primers employed in the RLM-RACE analysis, with denatured dsRNA from the 71 FCG-1609 strain as the template. This approach successfully amplified the terminal junctions for AlPV1 dsRNA1, dsRNA3, and dsRNA5 (Fig. 8A). Using inverse PCR, no amplicons was obtained for other dsRNA segments of AlPV1, or AlAV1, +ssRNA viruses and satellite-like elements (Fig. 8A). Sanger sequencing of the amplified fragments verified the 3′–5′ junctions; however, minor nucleotide deletions were observed (Fig. 8B). These results suggest that paticular dsRNA segments of AlPV1 may exist in circular or concatemeric forms in the host fungus.

AlBV1 has a bi-segmented dsRNA genome, similar to other orthocurvulaviruses (Tables 2, S1 and Fig. 3A). The 5′ ends are well conserved between the segments; however, the 3′ end sequence remains incomplete, even if it is likely to be more conserved than that of other members (Shahi et al., 2025) (Fig. S1C). BLAST analysis of AlBV1 RdRP (dsRNA1) identified Penicillium aurantiogriseum bipartite virus 1 as the top hit (68% amino acid identity). Phylogenetic analysis confirmed its placement in the genus *Orthocurvulavirus* (Fig. S3). According to the ICTV species demarcation criteria (≤85% RdRP identity), AlBV1 represents a novel viral species.

### 3.4 Identified +ssRNA viruses

Among the *A luchuensis* +ssRNA viruses identified, one was related to botourmiaviruses (*Ourlivirales*), two to narna-like viruses (*Wolframvirales*), and one to umbra-like viruses (*Tolivirales*). They were designated as Aspergillus luchuensis botourmiavirus 1 (AlBOV1), Aspergillus luchuensis narna-like virus 1 (AlNLV1), Aspergillus luchuensis splipalmvirus 1 (AlSPV1), and Aspergillus luchuensis umbra-like virus 1 (AlULV1) (Tables 2 and S1). The terminal sequences of AlBOV1, AlNLV1, and AlULV1, the terminal sequences were determined by RLM-RACE analysis (Fig. S1). Pairwise analysis of AlBOV1, AlNLV1, and AlULV1 ssRNA genomes revealed over 98% nucleotide sequence identity between the contigs of each virus.

BlastP analysis revealed that the RdRP of AlBOV1 shared 80% amino acid identity with Aspergillus flavus magoulivirus 1 (AfMoV, Table S1). Phylogenetic analysis placed AlBOV1 in the *Magoulivirus* clade alongside AfMoV1 (Fig. S4). Based on the ICTV criteria (<90% RdRP identity for distinct species) (Donaire et al., 2024), AlBOV1 and AfMoV1 are considered distinct novel species. Terminal sequence analysis identified two short contigs in strain 71 FCG-1609 (c54 and c23, ∼1 kb and 0.7 kb, respectively) with conserved 5′ and 3′ ends relative to AlBOV1 RNA1 (Fig. S1D). The 71 FCG-1609 contig (c53) contained a 5′ terminal region of over ∼120 bases, highly similar to that of AfMoV1 (93% identity). Both contigs sequences were detected alongside AlBOV1 RNA1, but some strains showed significant differences in the number of read counts (Fig. 6). These short RNAs are likely non-coding or encoding small proteins, and can be considered satellite RNAs of AlBOV1 (Fig. 3B), although homology with other sequences could not be confirmed. Additionally, a truncated polypeptide encoded by one contig in strain 71 FCG-1609 (c587) showed 36% amino acid sequence identity with the hypothetical protein of Chuzhou Botou tick virus 1 (CBTV1), a proposed botourmiavirus in the genus *Penoulivirus* possessing a unique genome structure that can code for the hypothetical protein downstream of RdRP. While the contig 71-FCG1609-c587 lacks the 5′ flanking region and terminal sequences, its co-occurrence with AlBOV1 and similar read count patterns suggests that it may represent a segment associated with AlBOV1 infection (Fig. 6).

**Fig. 9:**
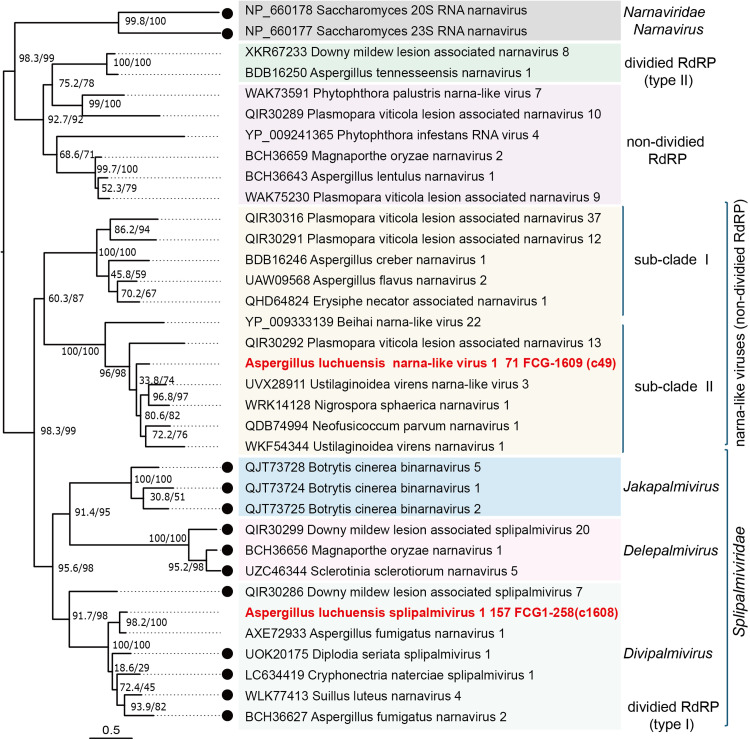
**Phylogenetic relationships of Aspergillus luchuensis narna-like viruses.** ML tree based on the RdRP alignment of narnaviruses, splipalmiviruses (type I-divided RdRP) and narna-like viruses (non-segmented subclades 1 and 2; segmented type II divided RdRP). The LG+I + G4 substitution model was used. Viruses identified in this study, Aspergillus luchuensis narna-like virus 1 (AlNLV1) and Aspergillus luchuensis splipalmivirus 1 (AlSPV1), are shown in bold and red.

Among the two narna-like viruses, AlNLV1 exhibits 56.5% RdRP identity with Neofusicoccum parvum narnavirus 1 (Table S1). Since 70% RdRP amino acid sequence identity was tentatively set as a species demarcation criterion for unassigned groups in this paper, AlNLV1 is considered a new species within the narna-like virus group. Terminal sequence analysis suggested a second RNA segment (RNA2), potentially encoding two ORFs (Figs. 3B, 6, and S1E). The first ORF protein shows 35.8% amino acid identity with a predicted protein from *Cuscuta epithymum* (a parasitic plant, Table S1). Phylogenetic analysis based on RdRPs placed AlNLV1 in subclade 2 of the narna-like viruses (Fig. 9). These results support AlNLV1 as a novel virus species; however, future taxonomic refinement is warranted. The second narna-like virus, AlSPV1, was detected only in strain 157 FCG-1258 and contained three RNA segments (Table S1 and Fig. 6). The putative proteins encoded by RNA1 and RNA2 shared 77.2% and 68.8% identity, respectively, with Aspergillus fumigatus narnavirus 1 (AfNV1) and Cryphonectria naterciae splipalmivirus 1 (CnSpV1). Its genome structure resembles that of splipalmiviruses, with a split RdRP domain encoded on RNA1 and RNA2 (Sato et al., 2023) (Fig. 3B). Phylogenetic analysis of RNA1 proteins placed AlSPV1 and CnSpV1 within the *Divipalmivirus* clade (Fig. 9). According to the ICTV species demarcation criteria (70% RdRP identity threshold; ICTV proposal 2024.003F.N.v1), AlSPV1 represents a novel species, alongside AfNV1 (for which only RNA1 sequence has been deposited in public database).

**Fig. 10:**
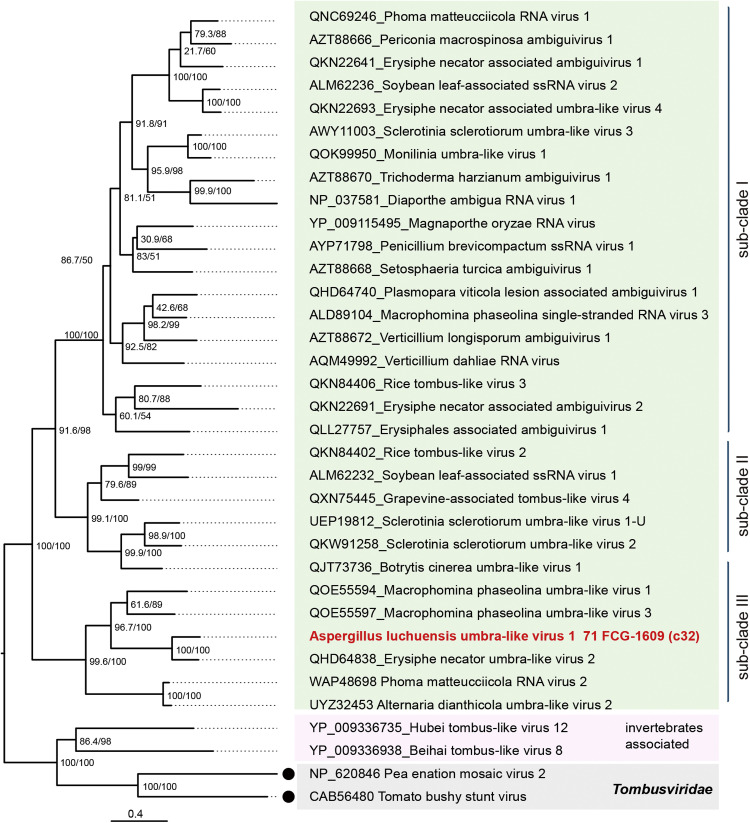
**Phylogenetic relationships of AlULV1.** ML tree based on the RdRP alignment of umbra-like viruses (order *Tolivirales*). The LG+F + I + G4 model was used. Two members of the family *Tombusviridae* (order *Tolivirales*) and two invertebrate-associated viruses (potentially from a related virus group) were used as outgroups.

AlULV1 encodes two ORFs and possesses longer 5′ and 3′ untranslated regions (UTRs) than other *A. luchuensis* mycoviruses (Fig. 3B). The RdRP encoded by the 3′ proximal ORF is predicted to be expressed as a readthrough protein. BlastP analysis showed 74.2% RdRP identity with Erysiphe necator umbra-like virus 2 (EnULV2) (Table S1). Phylogenetic analysis revealed three subclades among umbra-like viruses, with AlULV1 and EnULV2 forming subclade 3 (Fig. 10). Umbra-like viruses (order *Tolivirales*) remain unclassified and are also referred to as ambiguiviruses or mycotombusviruses (Ke et al., 2025; Zhou et al., 2023). AlULV1 and EnULV are considered to belonging the same novel species. A satellite-like RNA (∼0.5 kb) associated with AlULV1, with a relatively conserved 3′ end, was also detected (Tables 2, S1 and Fig. S1F). However, its low read count and limited detection across *A. luchuensis* strains (Fig. 6), its existence requires further confirmation.

## 4 Discussion

Research on mycoviruses has primarily focused on pathogenic fungi or mushrooms. However, viromes of a broader range of fungi have also progressed in recent years (Ghabrial et al., 2015; Ghabrial and Suzuki, 2009; Kondo et al., 2022). Mycoviruses in wine yeast, which are beneficial fungi used in fermentation, have long been recognized (Crucitti et al., 2022; Maske et al., 2022). Nevertheless, only a few reports have described mycoviruses in other fermentation-associated fungal species. For example, mycoviruses have been identified in *Neurospora intermedia* and *Aspergillus* spp., which are used in traditional fermented foods (to produce oncom and dried bonito [katsuobushi], respectively) (Buma et al., 2025, 2024; Honda et al., 2020). Viromic analysis has also been conducted for some food fermentation environments, including for cocoa and baijiu (Du et al., 2023; Maske et al., 2021; Santos et al., 2024; Zhang, 2025). This study focused on the viromes of an alcohol fermentation-associated *Aspergirillus* species (section *Nigri*), *A. luchuensis.* RNA-seq analyses revealed a diverse population of mycoviruses across multiple *A. luchuensis* strains isolated from local liquor production sites in Japan.

The family *Alternaviridae* currently comprises six recognized species, with many unassigned viruses infecting *Fusarium* spp. (Hua et al., 2024). Viruses related to AlAV1, including AfRV and Aspergillus heteromorphus alternavirus 1 (AhAV1), have also been identified in closely related species (*A. foetidus* and A. *heteromorphus*) within section *Nigri* from geographically distinct regions (Germany and Brazil) (Gilbert et al., 2019; Kozlakidis et al., 2013c). The German *A. foetidu* strain IMI 41,871 is co-infected with AfRV and a victorivirus (family *Pseudototiviridae*), and a capsidless +ssRNA yadokarivirus (*Yadokivirales*) (Kozlakidis et al., 2013a, 2013b), with the victorivirus acting as the helper virus for the yadokarivirus replication (Fadli et al., 2025). Distinct AlAV1 variants identified in samples from Okinawa, Hachijojima, and Aogashima Islands suggest potential geographic structuring of *A. luchuensis* mycoviruses (Fig. 1, Fig. 4). Similarly, *A. flavus* (section *Flavi*) isolates from northern Italy appear to harbor region-specific mycovirus communities (Degola et al., 2021), providing insights into host fungal population structure, dispersal, and colonization dynamics. Relatively closed, man-made fungal culture environments, together with their geographic isolation of the fungal samples collected in this study, may act as ecological islands that restrict horizontal gene flow between regions (Gallone et al., 2016). These conditions potentially promote the local evolution of both the host and its associated virome. Therefore, further analysis of the *A. luchuensis* virome, together with exploration of the host fungal population structure warrant future investigation. This information could help elucidate the local spread of domesticated fungi, the infection routes of mycoviruses and the fungal-virus co-evolution in these specific environments.

The genus *Gammapartitivirus* comprises three major subclades (Jaccard et al., 2023). Penicillium stoloniferum virus F (subclade I) is distantly related to other classified members (Nibert et al., 2014; Vainio et al., 2018) and multiple unassigned members, including AlPV1, constitute the third subclade (III, Fig. 5). Several partitiviruses possess dsRNA segments beyond two essential segments, dsRNA1 and dsRNA2 (Vainio et al., 2018). In deltapartitiviruses, the second and third segments encode CP and its homologues, whereas the function of the additional segments in other case remain largely unknown. Notably, a recent study of a *Drosophila* partitivirus identified a gene encoded by dsRNA4 that is involved in host male-specific lethality (male killing) (Kageyama et al., 2023). In AlPV1, additional segments closely related to dsRNA3 of other viral species (AfPV3 and BdV1) share ∼92% nucleotide sequence identity, whereas their dsRNA1 segments share 74%–76% sequence identity (Fig. 7), suggesting historical horizontal transfer (reassortment) between virus species from different *Aspergillus* sections or fungal families, as previously proposed (Petrzik, 2019; Telengech et al., 2020).

Recently, mycoviruses with circular RNA genomes have attracted increasing attention. These viruses possess ssRNA genomes and contain ribozyme sequences in their UTRs, as reported for ambiviruses and certain mycoviruses (Kuhn et al., 2024; Marquez-Molins, 2024). In addition, ribozyme or ribozyme-like sequences are present in the 5′ UTRs of mycoviruses with linear +ssRNA genomes (fusariviruses and hypoviruses) or dsRNA genomes (chrysoviruses and several other members of *Ghabrirale*) (López-Galiano et al., 2024). Although their precise function remains unclear, ribozyme or ribozyme-like sequences are presumed to be involved in RNA processing and/or translation (López-Galiano et al., 2024). The pseudo-circularization of the +ssRNA viral genome, known as the panhandle-shaped structure, has also been observed in flaviviruses (Nicholson and White, 2014). This process is mediated by base-pairing interactions between sequences in the terminal regions of the genome. Similarly, complementary sequences at the 5′ and 3′ ends of the bunyaviral –ssRNA genome can form a circularized panhandle structure (Malet et al., 2023). These structures are believed to contribute to the viral replication. Some dsRNA reoviruses are known to encode small non-coding circular RNAs, and they have a negative regulatory effect on viral replication (Pan et al., 2021; Shen et al., 2025). In this study, analysis of the partitivirus (AfPV1) suggested the presence of putative circular or dimeric (multimeric) RNA forms in certain genome segments; however, the presence or absence of ribozyme-like sequences remains unknown (Fig. 8). Similar observations have been reported for a dsRNA virus (a fungal chrysovirus), +ssRNA viruses (a mycovirgavirus and fungal endornaviruses) and –RNA viruses (plant orthotospoviruses) (Bertran et al., 2016; Hatvani et al., 2026; Horiuchi and Fukuhara, 2004). Although the mechanisms underlying the generation of such RNA molecules and their biological significance remain unknown, these forms may be advantageous for determining genomic terminal sequences as previously noted (Hatvani et al., 2026).

Satellite-like dsRNA elements, potentially non-coding, are present in some gamma- and other partitiviruses (Buma et al., 2024; Jiang et al., 2022; Oh and Hillman, 1995; Zhao et al., 2024). Their origin and distribution are poorly understood due to low sequence similarity. Interestingly, AfPV1 isolates harbor satellite-like dsRNA that can suppress helper virus accumulation and attenuate the reduction in pathogenicity of *A. flavus* (Jiang et al., 2022). Comparable effects have been reported in botybirnavirus satellite dsRNA and betapartitivirus defective dsRNA segment (Chiba et al., 2013; Ye et al., 2023). In AlPV1, although multiple dsRNA segments and satellite-like elements were detected, they were not consistently associated with virus isolates, suggesting their non-essentiality for viral replication (Fig. 6). Satellite-like RNAs were also observed i*n A. luchuensis* ssRNA viruses (AlBOV1 and AlULV1), consistently present in all AlBOV1-infected strains (Fig. 3, Fig. 6). Satellite-like RNAs or defective RNA have also been identified in association with several ssRNA mycoviruses, although their functions remain largely unknown (e.g. Hillman et al., 2000; Howitt et al., 2001; Li et al., 2019; Mizutani et al., 2018). Nevertheless, in *Pestalotiopsis fici,* a satellite-like RNA has been suggested to modulate helper ssRNA hypovirus accumulation (Han et al., 2023). These elements warrant further study to determine whether they are localized to specific regions, such as islands, or are more widespread.

Narna-like viruses (order *Wolframvirales)* were originally thought to possess non-segmented genomes. However, recent studies have revealed highly diverse architectures, including a divided RdRP-encoding genome and ambisense genomes (Chiba et al., 2021a, 2021b; DeRisi et al., 2019; Zhang et al., 2023). The newly discovered AlNLV1 exhibits a extra RNA segment (RNA2) encoding a hypothetical protein with moderate similarity to a parasitic plant protein (Table 2), suggesting possible horizontal gene transfer between mycoviruses and plants (Chiba et al., 2011). The RNA2 of AlNLV1 is consistently co-detected with RNA1, suggesting a potential essential viral function (Fig. 6).

Although dsRNA fractions were used as the template for RNA-seq, comparable reads were obtained for both ssRNA and dsRNA viruses (Fig. 2). However, replicative form (RF) dsRNAs of ssRNA genomes appeared to be largely undetectable on agarose gels (Fig. 1C), suggesting that they may possibly form higher-order structures or the undetectable RF dsRNA suffice as templates for cDNA synthesis. In *Cryphonectria* hypoviruses (a representative ssRNA mycovirus group), the accumulation of RF dsRNA varies among species. Cryphonectria hypovirus 1 readily accumulates detectable RF dsRNAs, whereas such accumulation is hardly observed for Cryphonectria hypovirus 4 (Aulia et al., 2019, 2021), highlighting differences in dsRNA accumulation profile and potential interactions with antiviral RNA interference.

In conclusion, we identified nine mycoviruses infecting *A. luchuensis* strains from a shochu brewing environment, at least five of which represent novel species. The unique features of these viruses,including the dynamics of genomic segments, satellite-like RNA elements, and potential reassortment or horizontal gene transfer, highlight their biological and evolutionary significance. These findings reveal the presence of mycoviruses in fermentation-associated species and provide new insights into the diversity, dynamics, and population structure of *Aspergillus*-infecting mycoviruses on geographically specific islands. Considering the economic importance of *A. luchuensis* or other *Aspergillus* species in alcohol fermentation and food processing, whether mycoviruses have an impact on the phenotypic and physiological characteristics of their fungal hosts, particularly those related to their industrial use, warrants investigation.

## Acknowledgements

We thank Dr. Yuji Kawakami (the FCG Research Institute, Inc.) and the RIKEN BioResource Research Center for kindly providing fungal strains. We would like to thank Sotaro Chiba, Mayuna Shiba, Muhammad Fadli, Kazuyuki Maruyama, Sakae Hisano, Hideki Nishimura, and Takakazu Matsuura for their helpful technical assistance or fruitful discussion. We also thank the handling editor and three reviewers for their assistance and valuable suggestions.

## Data deposition

RNA-seq data have been deposited in the DDBJ/EMBL/GenBank databases under the accession numbers, PRJDB40404 (BioProject) DRR909486-DRR90949 (DRR Run). The nucleotide sequences of *A. luchuensis* viruses have been deposited with the DDBJ/EMBL/GenBank databases under Accession Nos. LC923879-LC923914.

## Funding

This work was partly supported by the KAKENHI program of the Japan Society for the Promotion of Science (grant numbers 20K05791 [to FF), 23H02214 and 23K18029 [to HK and NS], the Joint Usage/Research Center, Institute of Plant Science and Resources, Okayama University, and the Ohara Foundation for Agriculture Research (Kurashiki, Japan).

## CRediT authorship contribution statement

**Hideki Kondo:** Writing – review & editing, Writing – original draft, Visualization, Validation, Supervision, Methodology, Investigation, Funding acquisition, Formal analysis, Data curation, Conceptualization. **Misaki Nanaji:** Validation, Investigation, Formal analysis, Data curation. **Hitomi Sugahara:** Validation, Investigation. **Miki Fujita:** Validation, Investigation. **Ida Bagus Andika:** Writing – review & editing. **Nobuhiro Suzuki:** Writing – review & editing, Supervision, Funding acquisition, Conceptualization. **Fujimori Fumihiro:** Writing – review & editing, Validation, Supervision, Resources, Investigation, Funding acquisition, Conceptualization.

## Declaration of competing interest

The authors declare that they have no known competing financial interests or personal relationships that could have appeared to influence the work reported in this paper.

## Data Availability

Data will be made available on request.

## Appendix Supplementary materials

Image, application 1
