# Supplementary material for: Mycoviruses diversity in the black kōji mold, Aspergillus luchuensis (section Nigri) isolated from liquor-production environments in Japan
Source: Virus Res. 2026 Apr 5;367:199724. doi: 10.1016/j.virusres.2026.199724 (PMC13122248; doi:10.1016/j.virusres.2026.199724)
Supplement: Supplementary file 1 [file mmc1.pdf]

Supplementary information for:

Virus Research SI: Virosphere of the kingdom Fungi

Mycoviruses diversity in the black kōji mold, *Aspergillus luchuensis* (section *Nigri*) isolated from liquor-production environments in Japan

Hideki Kondo[1,*], Misaki Nanaji[2], Hitomi Sugahara[1], Miki Fujita[1], Ida Bagus Andika[3], Nobuhiro Suzuki[1,4], Fujimori Fumihiro[2,*]

[1]Institute of Plant Science and Resources, Okayama University; Chuou 2-20-1, Kurashiki, Okayama 710-0046, Japan

[2]Graduate School of Tokyo Kasei University, 1-18-1 Kaga, Itabashi, Tokyo 173-8602, Japan

[3]Northwest A&F University, Yangling, China

[4]Graduate School of Agricultural Science, Tohoku University, Sendai, Japan.

Table S1. Virus-like contigs assembled from the dsRNA samples of *A. luchuensis* strains

virus	segments	size (nt)	dsRNA seq	Average coverage	Accession	BLAST-P result (top hits)			
			Contig name			virus_segment No.	protein*	Identity	Accession
dsRNA viruses									
Aspergillus luchuensis alternavirus 1 (AlAV1) variant A	dsRNA1	3571	71-FCG1609_ c43	14033	LC923879	Aspergillus foetidus dsRNA mycovirus, seg 1	RdRP	97.8%	YP_007353985
	dsRNA2	2737	71-FCG1609_ c255	4807	LC923880	Aspergillus foetidus dsRNA mycovirus, seg 2	HP	98.2%	YP_007353982
	dsRNA3	2424	71-FCG1609_ c27	5087	LC923881	Aspergillus foetidus dsRNA mycovirus, seg 3	HP	97.4%	YP_007353983
	dsRNA4	1951	71-FCG1609_ c38	4711	LC923882	Aspergillus foetidus dsRNA mycovirus, seg 4	HP	45.2%	YP_007353984
variant B (B1)	dsRNA1	>3537	JCM22320_c41	20497	LC923883	Aspergillus mycovirus 341	RdRP	98.0%	ABX79997
	dsRNA2	>2758	JCM22320_c7	21417	LC923884	Aspergillus foetidus dsRNA mycovirus, seg 2	HP	98.3%	YP_007353982
	dsRNA3	>2418	JCM22320_c13	38143	LC923885	Aspergillus foetidus dsRNA mycovirus, seg 3	HP	98.5%	YP_007353983
	dsRNA4	>1920	JCM22320_c4	10906	LC923886	Aspergillus foetidus dsRNA mycovirus, seg 4	HP	45.8%	YP_007353984
variant C	dsRNA1	>3500	58-FCG1259_ c445	488	LC923887	Aspergillus mycovirus 341	RdRP	92.9%	ABX79997
	dsRNA2	>2111	58-FCG1259_ c133	267	LC923888	Aspergillus foetidus dsRNA mycovirus, seg 2	HP	94.9%	YP_007353982
	dsRNA3	>2235	58-FCG1259_ c176	269	LC923889	Aspergillus heteromorphus alternavirus 1, seg 3	HP	95.7%	AZT88577
	dsRNA4	>1921	58-FCG1259_ c1333	127	LC923890	Aspergillus foetidus dsRNA mycovirus, seg 4	HP	77.4%	YP_007353984
Aspergillus luchuensis partitivirus 1 (AlPV1)	dsRNA1	1760	71-FCG1609_ c15	5284	LC923891	Aspergillus niger partitivirus 1, seg 1	RdRP	94.3%	BDF97659
	dsRNA2	1378	71-FCG1609_ c13	30799	LC923892	Aspergillus niger partitivirus 1, seg 2	CP?	96.3%	BDF97658
	dsRNA3a	1188	71-FCG1609_ c164	9426	LC923893	Aspergillus flavus partitivirus 1, seg 3	HP	93.2%	QDE53636
	dsRNA3b	>1108	71-FCG1609_ c231	8001	LC923894	Aspergillus flavus partitivirus 1, seg 3	HP	93.2%	QDE53636
	dsRNA4	>1126	71-FCG1609_ c68	12603	LC923895	Botryosphaeria dothidea virus 1, seg 3	HP	88.3%	AIE47696
	dsRNA5	771	71-FCG1609_ c14	35088	LC923896	no hits	-	-	-
	dsRNA6	693	68-FCG1606_ c11	62144	LC923897	no hits	-	-	-
Aspergillus luchuensis partitivirus 2 (AlPV2)	dsRNA1	>1878	JCM22320-c5	89475	LC923898	Colletotrichum eremochloae partitivirus 1	RdRP	62.0%	AZT88590
	dsRNA2	>1713	JCM22320-c3	86445	LC923899	Penicillium aurantiogriseum partiti-like virus 1	CP	53.7%	ASY04022
Aspergillus luchuensis partitivirus 3 (AlPV3)	dsRNA1	>1764	71-FCG1609_ c816/c92357**	12	LC923900	Helicobasidium mompa partitivirus V1-1	RdRP	63.1%	BAD32677
	dsRNA2	>2079	71-FCG1609_ c1758	38	LC923901	Cucurbitaria piceae virus 1	CP	37.2%	ALT08066
Aspergillus luchuensis bipartite virus 1 (AlBV1)	dsRNA1	2106	68-FCG1606_ c4	13054	LC923902	Penicillium aurantiogriseum bipartite virus 1	RdRP	68.0%	YP_009182335
	dsRNA2	1856	68-FCG1606_ c37	11453	LC923903	Curvularia thermal tolerance virus	HP	63.3%	YP_001976145
ssRNA viruses									
Aspergillus luchuensis botourmiavirus 1 (AlBOV1)	RNA1	2845	71-FCG1609_ c166	5284	LC923904	Aspergillus flavus magoulivirus 1	RdRP	79.9%	UAW09569
	sat-like 1	>965	71-FCG1609_ c54	36415	LC923906	no hits	-	-	-
	sat-like 2	>704	71-FCG1609_ c23	66823	LC923907	no hits	-	-	-
	(RNA2?)	>987	71-FCG1609_ c587	2961	LC923905	Chuzhou Botou tick virus 1	HP	36.0%	UYL95456
Aspergillus luchuensis narna-like virus 1 (AlNLV1)	RNA1	3138	71-FCG1609_ c49	1496	LC923908	Neofusicoccum parvum narnavirus 1	RdRP	56.5%	QDB74994
	RNA2	1273	71-FCG1609_ c271	1023	LC923909	*Cuscuta epithymum* (a parasitic plant gene)	HP	35.83%	CAH9129646
Aspergillus luchuensis splipalmivirus 1 (AlSPV1)	RNA1	>2049	157-FCG1258-c1608	52	LC923910	Aspergillus fumigatus narnavirus 1, seg1	RdRP	77.2%	AXE72933
	RNA2	>2279	157-FCG1258_ c603	87	LC923911	Cryphonectria naterciae splipalmivirus 1, seg 2	HP	68.8%	UYL95385
	RNA3	>1412	157-FCG1258_ c136	518	LC923912	Grapevine wood holobiome associated narna-like virus 13	HP1	63.2%	XOD45370
Aspergillus luchuensis umbra-like virus 1 (AlULV1)	RNA1	3751	71-FCG1609_ c32	36925	LC923913	Erysiphe necator umbra-like virus 2	RdRP	74.2%	QHD64838
	sat like	>498	71-FCG1609_ c338	4938	LC923914	no hits	-	-	-

*: RdRP, RNA-dependent RNA polymerase; HP, hypothetical protein; CP, capsid or coat protein

**: a concatenated sequence from two contigs (c8167/c9235) with a gap-reasion.

A.

1.Aspergillus luchuensis alternavirus 1 (AlAV1) variant A_71 FCG-1609

[5’-Ter]

```
AlAV1-1 c43  gtggctgacagtccg-gtttggaactcattcgagtacccatccgctgtgcgtgatgcgaa
AlAV1-2 c255 ttggctgacagtccgtgtttggatctcattcgagtaccatccggctgtacatgatgcttt
AlAV1-3 c38  ttggctgacagtccg-gtttggagtacagtcaagtacccatccgctgtgcatgatgctgt
AlAV1-4 c27  ttggctgacagtccg-tagtggagtagattcaactacccacacgctgtacaagatgcttt
              **************    **** .  * **.* ****  .  *****.*. *****

AlAV1-1 c43  ---------------gcgttcagatagt-----------------gctggaaaatgcgcg
AlAV1-2 c255 ---------------------------------------------gcttcaacgttctcg
AlAV1-3 c38  ---------------------------------------------gcgttaacattcgcg
AlAV1-4 c27  cgaatgaggagtttgctgccaagatggaggctcggaggaacctcgccttggagatgctag
                                                           *   .* .* *  *
```

[3’-Ter]

```
AlAV1-1 c43  atgtttcggcacgctttcccgcatttattc-ttgcattagctgggtcacgcgatctcctt
AlAV1-2 c255 acgctgagctacgaaaactcg-----------tgggctagctgggtcatgtaatctcctt
AlAV1-3 c38  acgct--------------------------------ctgctgggtcatgcaatctccta
AlAV1-4 c27  gggtcgtgctatgggagcttacatttggtcatcattctagctgggtcttgtaatctccta
             . *..                                . ******** .*..*******

AlAV1-1 c43  tgttcaatcattaaaccactgagtgtgaggctacatccctga--gtc-ctcaatgggtag
AlAV1-2 c255 tgtctattctctata--------tgcgatgctggatccccacctacg-tgtgatggccag
AlAV1-3 c38  ttgttaacctccata-------aagcgatgccggatccccac--atg-cgtgatggccgg
AlAV1-4 c27  tgttta-tcattata--gctatatgcgaggctggatccccacttatatcgtgatggccag
             *  ..* .* ..* *         *.** **.. *****..   ..  . ..**** ..*

AlAV1-1 c43  ctagcac-----------------------------------------------------
AlAV1-2 c38  ctagcaacggagcgtgccgtacacgccagcagtgtccgggaataaggccgagctgtggtt
AlAV1-3 c38  ctagcacgggagc-tgcccttatcgccagcagtgtccaggaataaggctgagctgtggta
AlAV1-4 c27  ctagctacggagcgtgccgtacacgccagcagcgtccaggaataaggctgagctgtggat
             *****

AlAV1-1 c43  ----gcctgaggtcagatccctagtaggtgagtactaatggctgactaaggtctgtttac
AlAV1-2 c255 taaagtcatatggtggatccctgatgggtgagcatcaatggcca-------------cat
AlAV1-3 c38  tatagtcatatggtggatccctggtgggtgagcaccaatggcca-------------cat
AlAV1-4 c27  taaagtcatatggtggatccctggtgggtgagcaccaatggcca-------------cat
                 *.*  * * ..*******..*.******.*..******..             .*.

AlAV1-1 c43  ttttaaa(poly-A)
AlAV1-2 c255 gtctgac(poly-A)
AlAV1-2 c38  gtctgac(poly-A)
AlAV1-4 c27  gtctgac(poly-A)
              *.*.*
```

B

2. Aspergillus luchuensis partitiviru 1 (AlPV1)_71 FCG-1609

[5’-Ter]

```
AlPV1-1  c15  ttacttttgtctgcacaggttgtagtgggcctgactcaatgagt------aagacatctg
AlPV1-2  c13  gtacttttatc--ctgaagttctattggtacgaacgaaacttacgttttaaggatatctg
AlPV1-3a c164 taacttttgtt--cttaaaagctattggtacgcttgcgatccagttt----gaacatctg
AlPV1-4  c68  -aacttttgat--cttgag-tctattggtacgactacacttcggtgg---agatcatctg
                ******. .  *  ...   ** ***  *   .  . .  .        .. .*****

AlPV1-1  c15  aacccttcaccgcctttca-----------atttgattaccccggaaagagcgacgttgt
AlPV1-2  c13  aaccctgcctccaaact---------------------------------attagacacc
AlPV1-3a c164 aaccttcgatctaacctcg-----------aaatactgcagccaatacccccagtatatt
AlPV1-4  c68  aacccagttgcgttacgggtcgcggacgcctactgctacctgtgatttatactgtacact
              ****.     *    .                                   . . ..  .
```

[3’-Ter]

```
AlPV1-1  c15   ggacacacctcgtcca---cgcctagtc--tcgatcgagagcccctcattcaa--tctca
AlPV1-2  c13   gctagtgccccgcc-----ggttaaggt--gccaacgaagatttcttcttcaatctctat
AlPV1-3a c164  gtgagtgttgtgctcacagggactagtcggtcgatgtggcgtttaacagacaaaacttaa
               *   ..... .*..      * . ** .   * *   .. ....  .   ***  ..*

AlPV1-1  c15   tctcctcgattca
AlPV1-2  c13   ctcccttaactca
AlPV1-3a c164  taccettgaccca
               . .***..*..**
```

Aspergillus luchuensis partitiviru 1 (AlPV1)_71 FCG-1609 dsRNA5

[5’-Ter]

```
AlPV1-5 c14    aaactttt----------------------------------------------------
AlPV1-3a c164  taacttttgttcttaaaagctattggtacgcttgcgatccagtttgaacatctgaacctt
                *******

AlPV1-5 c14    -------------aagttgcttatgcgacaccccccatcggggttctaaagatgggcagg
AlPV1-3a c164  cgatctaacctcgaaatactgcagccaatacccccagtatattttcc----ctccgcata
                       **.*  . .*  *.*.****** .*  .  ***.     *  *** .

AlPV1-5 c14    ttctagagattcctgtaagtttgaa
AlPV1-3a c164  ggcgagagatgcatgttgattgcaa
                 * ****** * *** ..**  **
```

[3’-Ter]

```
AlPV1-3a c164  c------ctatgatttagta--------taaaaatatttgctttcaaacccagtacgccg
AlPV1-5 c14    cggtactaaatgactcgcaacatcatgttaaagatgtgaccgcgcagatcaaacacgatt
               *        ****.*..  *        ****.**.*   * . **.*.* *..*** .

AlPV1-3a c164  aaagacggaactgtgagtgttgtgctcacagggactagtcggtcgatgtggcgtttaaca
AlPV1-5 c14    aacgatcaa---------------------------------------------------
               ** **. .*

AlPV1-3a c164  gacaaaacttaatacccttgaccca
AlPV1-5 c14    -------ctatattcctttgattca
                      **  ** **.****..**
```

Aspergillus luchuensis partitiviru 1 (AlPV1)_68 FCG-1606 dsRNA6

[5’-Ter]

```
AlPV1-5 c14    -aaacttttaagttgcttatgcgacaccccccatcggggttc---------taaagatgg
AlPV1-6 c11    caaactttaaac--acttatgtgtgcc--ccccgcggggttacttcggtaataaag----
                ******* **    ****** *   *  ***  *******          *****

AlPV1-6 c11    gcaggttctagagattcctgtaagtttgaaatacatcagt
AlPV1-6 c11    -cggctaggaatgatgcctagaagtttaaaagg-------
                * * *   *  *** ***  ****** ***
```

[3’-Ter]

```
AlPV1-5 c14    TACTCGGTACTAAATGACTCGCAACATCATGTTAAA-------G--------ATGTGACC
AlPV1-6 c11    -------------------------CTCATGTTGTGACTCTTGGGTGATACCAAGACGCC
                                         *******          *        * *   **

AlPV1-5 c14    GCGCAGATCAAACACGATTAACGATCAACTATATTCCTTTGATTCA--------------
AlPV1-6 c11    TCGCAAGT-TTCTACGATTATCGATCAAAT-TATTACATACACTGAAACTCGTAAATGGA
                ****  *     ******* ******* * **** * *  * * *

AlPV1-5 c14    -------       91
AlPV1-6 c11    GGAGGCC       100
```

3. Aspergillus luchuensis partitivirus 2 (AlPV2)_JCM 22320

[5’-Ter]

```
AlPV2-1_c5    --CATAAAGCTATCGTTTTATGCTATTGTTACTTGGTGAAAGAGAATTCTCGTAAGAATC
AlPV2-2_c3    CCCAAAAACCTGTGGATATTCG-T--ATTTCCTTCGGGAACAAAAACT--CCATAGAGGT
                ** *** ** * * * *  * *    ** *** * ***  * ** *  *   ***

AlPV2-1_c5    TTAAATCTTCTATCACCATGGTAGCGTTACGGCACCTCAAACGGGTATCCC---------
AlPV2-2_c3    TTAAATACT---------TGGTAGTTTA----TAAGTCGATCTGGTATCGCAACAATCAA
              ******  *         ******  *      *  ** * * ****** *
```

[3’-Ter]

```
AlPV2-1_c5    GTGAAACACGTTTTACTGATTTTCATAAATATACCCAGACGTTCGTTTCTCGCTACTCAT
AlPV2-2_c3    GT-------------------TATATAAAATTACAAAAACAATTTAAAATT--TATAAAA
              **                   *  *****  ***  * **  *      *   **   *

AlPV2-1_c5    TTGATGATGATGAGAGGTTCTTTTCTGATTAATTGTTCGGCAATTGGTGTTC-CACAGAT
AlPV2-2_c3    TTTAG---AAAAAGAAGTGTT--------TATGGGTTGGCGACCTTATCTAGCCAAGGAC
              ** *     *  *** **  *        **   *** *  *  *  * *   **  **

AlPV2-1_c5    CGGAAGAGCGTC
AlPV2-2_c3    GGGCTGAC-G--
               **  **  *
```

4. Aspergillus luchuensis partitivirus 3 (AlPV3)_71 FCG-1609

[5’-Ter]

```
AlPV3-1_c8167  -------------GCCTCCTTCTTATAGTTTTATACTAAAATCACATTTCTCCAAA----
AlPV3-2_c1758/ CAGGCCCCCCCACGCCTCCTTCTAAAAT---TTTACAACTATCACATTTCTCCAATTTAC
         1578               ********** * *    * *** *  ***************

AlPV3-1_c8167  AACATTCACTATGTTCAACCA--AGTTAGAGATTATCT----CTCTGAGAAAGTCTCCTA
AlPV3-2_c1758/ AATCTTAACAATGTCTTCTCGGTCTTTTGATGTTTCTTCACGCGTTACGAAAAT----TA
         1578  **  ** ** ****     *     ** **  **   *    *  *  **** *    **
```

[3’-Ter]

```
AlPV3-1_c8167  TCGCTATCATATCGAGTTAACTATGGAAGACCAACACGCCCAATTGGCAAGCTAGTTGCA
AlPV3-2_c1758/ TCACTAATTT-CAGAACCATCTTTGGAACTG--A--CG--TTACTATGACTGAAGTTTCA
         1578  ** ***   *   **   * ** *****     *  **    * *   *    **** **

AlPV3-1_c8167  CAACTATGCTATCCTGAACGTG-------------GAC---
AlPV3-2_c1758/ CA------TTATCTTGAAGCCATGCCAATCATCTGAATCAC
         1578  **       **** ****                  *
```

C

5. Aspergillus luchuensis bipartite virus 1 (AlBV1)_68 FCG-1606

[5’-Ter]

```
AlBV1-1 c4   AGCCACCGGAACTTAAGTACTTGTCCCCAGACGACTTTCCAACGACAGTGAAACATCTCA
AlBV1-2 c37  -ACCACCGGAACCTAAGTACTTGTCCCCAGACGACTTTCGACAAACAATCAAAGATATCA
               ********** ************************** *   *** * *** ** ***

AlBV1-1 c4   TTGAGATATCATATAAATCGAACATCGCAACACGACGCAAGACAACTTTCGACACCAACG
AlBV1-2 c37  CCGAGGTATCAGTCTAGCCGAACTTCACACGACCTTGCAAGCACGCTTCGACACGCCCCG
               *** *****    *  ***** ** **  **   *****    ***       *  **
```

[3’-Ter]

```
AlBV1-1 c4   TGAGCGTTCTCCTGTATTTAGAGTTC--CATCCTGTTTGCGCAGGGTGAAGACTGGGTCC
AlBV1-2 c37  TGTTTGGCAGCATGTATGTAGAGTTCCCGGGTGGCGCAAACCAACCGGAAGACTGGGTCC
             **   *    * ***** ********               **    *************

AlBV1-1 c4   GAGTTCAGTGGCTGGCAGGCGAGCCATCCTCGGGGGAAACAGTTACCTTATCAAATCGAG
AlBV1-2 c37  GAGTTCAGTGGCTGGCAGGCGAGCCATCCTCGGGGGAAACAGTTACCTTATCAAATC---
             *********************************************************

AlBV1-1 c4   CCGTT
AlBV1-2 c37  -----
```

D

6. Aspergillus luchuensis botourumiavirus 1 (AlBOV1)_71 FCG-1609

[5’-UTR]

```
AlBOV1_c166     tggtctgttggaccaaaacgccctagggcgttttgctctgcagacactggctcgaaag--
AlBOV1_c23(sat) tggtctgtttggccaaaatgccctagggcatcttgtgcaacag-cctggccaaggtgg--
AlBOV1_c54(sat) tgatctg-tagatcaaaacgccttagggcgtcttgctcagcagatactggcctgattggc
                **.**** * *..*****.***.******.*.***. * .*** . . * *  *.  *

AlBOV1_c166     cctacgtctcagttattagagacggatgagataacatcaagtccactcgaacgcgagtc-
AlBOV1_c23(sat) cctacaactcagttattagagtcggatgagataacatcaagtccacttaaacgctgggt-
AlBOV1_c54(sat) catacgatcgagttattacgatcggcttcggtcgaaacggatatcttgggtcgtgtggtc
                * ***. .. ******** .. *** *  *.* . * *...* . .* .. **.  * .

AlBOV1_c166     ------------gcgggttattagagaaagccagaaccactggc--------------ta
AlBOV1_c23(sat) ------------acg---ttggaagctttggaagggcttcggcc--------------ta
AlBOV1_c54(sat) acccagctaccgacggaatttgagggcccgtcagcccgatctgcacgttagtggttggca
                            .**   *   *..    *  **  *  .   *              .*

AlBOV1_c166     agaga------------------------------cggtaagagcgccgtcccacgcat-
AlBOV1_c23(sat) gaatac----------------------------tcggtaagagcgccgagtcgc-----
AlBOV1_c54(sat) ggaaaccggcatttgaaaagaatggcggcaaatcacagtaagaacgctgtgttgcatgtt
                ..* *                              *.******.***.*  ...*

AlBOV1_c166     ------------------------------------------------------------
AlBOV1_c23(sat) ------------------------------------------------------------
AlBOV1_c54(sat) ctcagagcatgtcaaggaggatggcattgagggtagtccgggccgcggccgcggtacccc


AlBOV1_c166     ------------------------------------------------------------
AlBOV1_c23(sat) ------------------------------------------------------------
AlBOV1_c54(sat) tcgatagtcaatgtaaaatcgacttcggtcgttgtgggatctaaaagcttcccagcctcc


AlBOV1_c166     -------------------gtcatttgtttggctgccgggctaagaactacagctcatca
AlBOV1_c23(sat) -----------------------tacgtttggttggagg----tgatcagcctctcatca
AlBOV1_c54(sat) gcccattcctcgctttgagattttctaggttgtcggggg----tcgtcagcccctcatca
                                       * ..  * *..*  **      . * .*  *******

AlBOV1_c166     gtctttcaggtgatgataacc-tacccgtggtgatggggtccacatagaggcccccgtat
AlBOV1_c23(sat) gtctttctggtgatgataaccattgatcgaacgatagtgtctacat--------------
AlBOV1_c54(sat) gtctttctggtgatgataaccaatgacggaatgaaagtgcatcgta--------------
                ******* *************     .  ...** .* *. .
```

E

7. Aspergillus luchuensis narna-like virus 1 (AlNLV1)_71 FCG-1609

[5’-Ter]

```
AlNLV1-1_c49      -----acgagtcaatatggactcaattcgctgggcacccccacttccgaccatcggccgg
AlNLV1-2-c271     acgaccgaagtcaaatcgcgatcaatccacgtattgatcgtacttccgctc-ccgcacaa
                         .******  .* . *****.*.*  . .. .* .******* .* .**  *..

AlNLV1-1_c49      gcggggcccagaatcgtcgtgctacctagtagca
AlNLV1-2-c271     acaggtc---gattccccgtttaaggaatcaaca
                  .*.** *   ** ** .*** . *   * .*.**
```

[3’-Ter]

```
AlNLV1-1_c49      atgcggagatccgcatcgaacacacccgttgggagggtgtgattgtggctaaaatatagt
AlNLV1-2-c271     ---cggactagagagctatgctctctctctctgctgatacagcggaggc-----------
                     ****     * .... .* * *.* .*  *  *.*..... * ***
```

```
AlNLV1-1_c49       acaatgtgcatcttagatgtacatcac--agtaagccaaaactggcactgcgattctgg
AlNLV1-2-c271     -------cacatcttagatgtggactaccaagctcaccaaaattggggcttggcgaccgg
                         ..************. *..**  **.  .******.*** .**  *   *.**

AlNLV1-1_c49      gctcaagcccgt
AlNLV1-2-c271     gtcttaacccgt
                  *... *.*****
```

8. Aspergillus luchuensis splipalmivirus 1 (AlSPV1)_157 FCG-1258

[5’-Ter]

```
AlSPV1-1_c1608       -------GTCTCGTCTTTCGACGGATTTATCC-CTGATTACGGGATCTAGAGGGTTCAAT
AlSPV1-2_c603        CTTTCGGGTCTCGTCTTTCGACGGATTTAATCCTTGATTACGGGACTTAGAGGGTTCAGC
AlSPV1-3_c136        CTTTCGAGTCTCGTCTTTCGACGGTATTAGCG-AGAGT----------------------
                            *****************  ***        *
```

[3’-Ter]

```
AlSPV1-1_c1608       TCGCACAACGCAGGTTAGACCAGGGAAAA--CCTGGTACTCCTACGGCCGAGAGGCCAGA
AlSPV1-2_c603        TCGCGCAAAGCAGGTTAGATAGTGGGAAAACCACTATACTCCTACGGCCGCAAGGCCAGA
AlSPV1-3_c136        TAAC--------------------GAAAC-------------CCCGGCCGACAGGCCACA
                     *  *                    * **                ******  ****** *

AlSPV1-1_c1608       CTCACA-----
AlSPV1-2_c603        CTCATCCGAGC
AlSPV1-3_c136        CCGCATCCGGT
                                  *
```

9. Aspergillus luchuensis umbra-like virus 1 (AlULV1)_71 FCG-1609

[5’-Ter]

```
AlUlV1 c32        tggggaaaacatgtgaggtaggatagcatgttttctgtcggaagcgtcctacgaggctcc
AlUlV1 c-338(sat) gtgcgaa------------------------------ccagtcgcgtagcagggttttca
                    * ***                              .*.*  ****  .* *.  .**

AlUlV1 c32        atccattccgggtagctcggcccggtggcgtcctgaggagcagcgcagtggattcgcccg
AlUlV1 c-338(sat) agcgttgtcgtactgctagccttgggtgcctaaccccgaggtgaagggtggctgcg----
                  * *  * .** .. *** * *..**  ** *  .   ***  * . .**** * **
```

[3’-Ter]

```
AlUlV1 c32        tttaaaaatcttcggagggcggaac--------------agcgtcttaactgtctcaaga
AlUlV1 c-338(sat) -------actttggggagatggcacttatcccctcagttctcttctgtgcagccccaaga
                         *..** **..*..** **                * ***  .* *.*.*****

AlUlV1 c32        ttcgccttcgggccgcccca
AlUlV1 c-338(sat) ttcgccttcgg---------
                  ***********
```

Fig. S1. Multiple alignment of 5' and 3' terminal regions of viral contig sequences. The MAFFT platform was used to align two or more sequences. For RLM-RACE analysis, the version amplicon or its cloned fragment directly sequenced. The results are presented as a CLUSTAL format alignment by MAFFT (version 7.503).

Alternaviruses_RdRP (amino acid sequences)/ ML tree

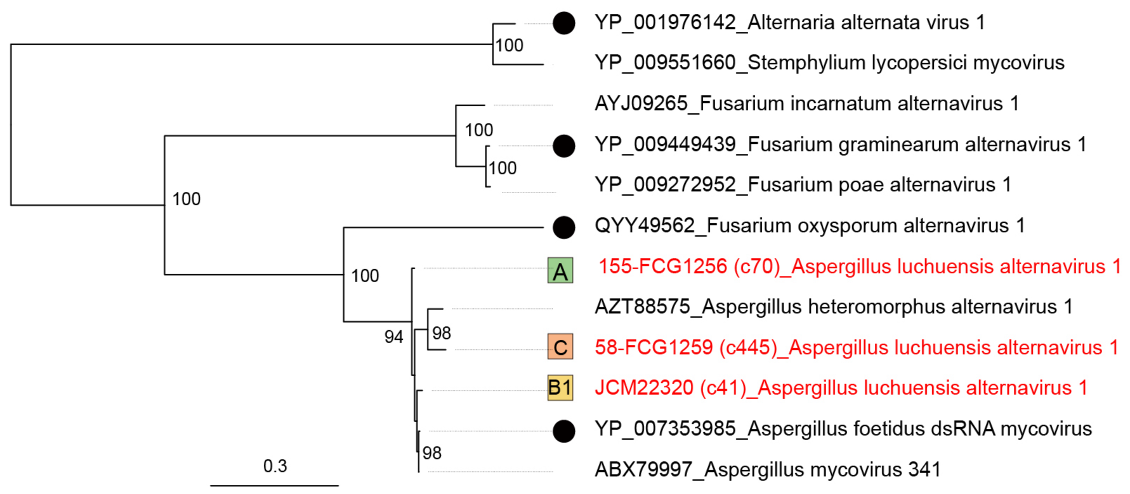

Fig. S2. Phylogenetic relationship of Aspergillus luchuensis alternavirus (AlAV1) variants within *A. luchuensis* strains. The ML tree was constructed based on the RdRP alignment of AlAV1 variants and other altenraviruses. The replisentative virus isolates of AlAV variants (A, B1 and C) and the established Alternavirus species are indicated by boxes and filled circles, reespectively.

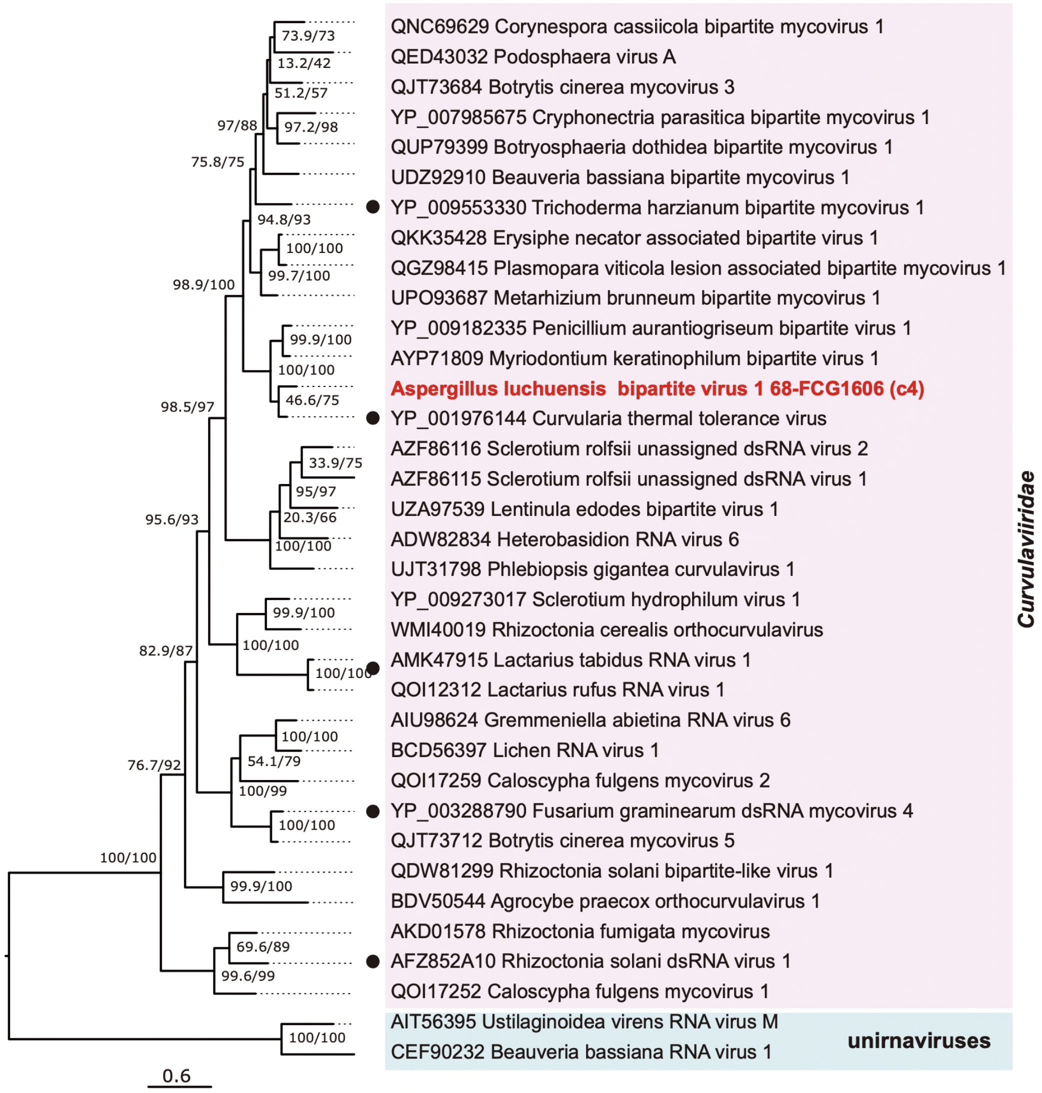

Fig. S3. Phylogenetic relationships of Aspergillus luchuensis bipartite virus 1 (AlBV1). ML tree based on the RdRP alignment of curvulaviruses (family Curvulavridae, order Durnavirales) and their relatives. The rtREV+F+I+G4 substitution model was used. Two unirnaviruses (family Amalgaviridae) were used as outgroups.

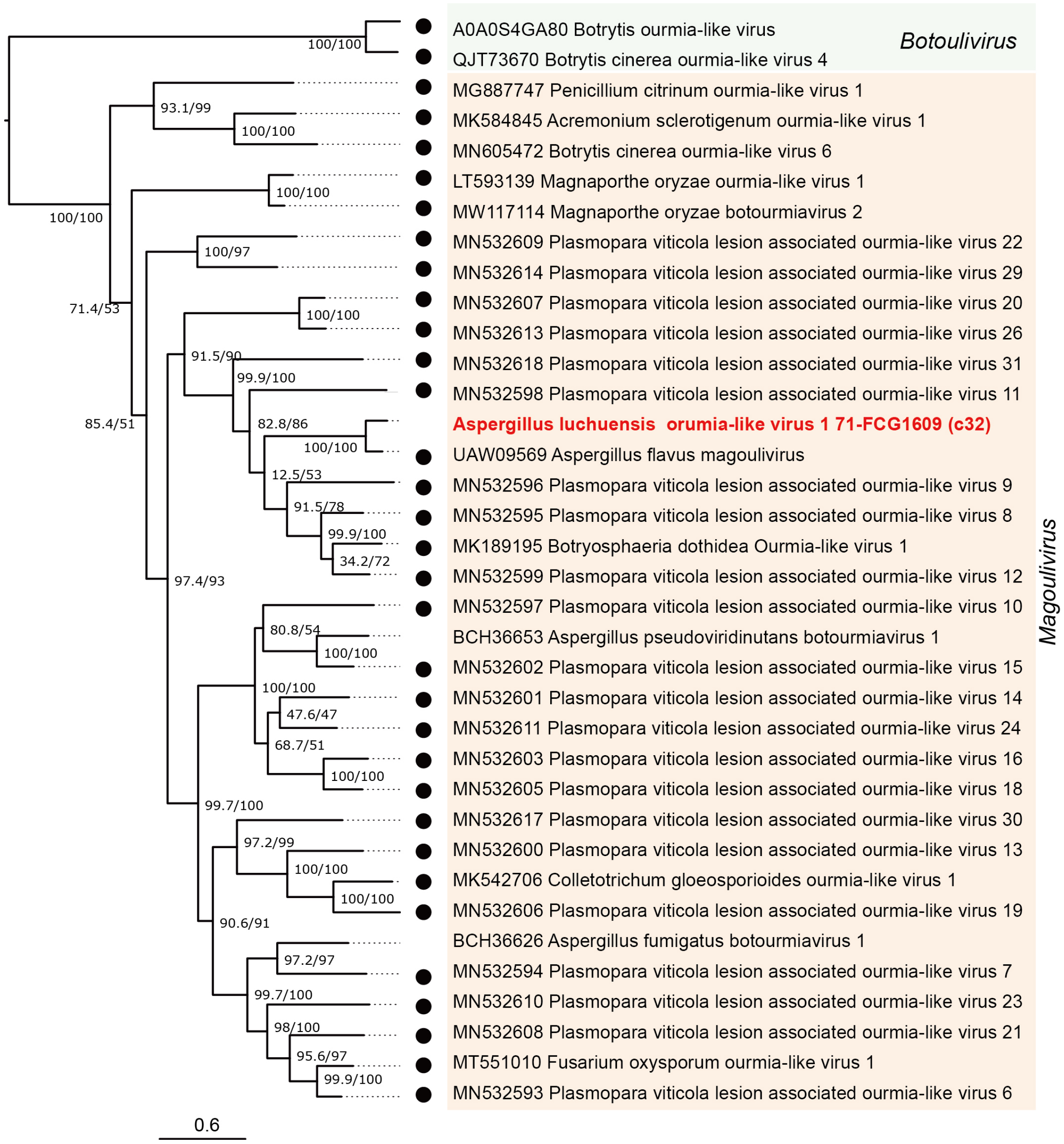

Fig. S4. Phylogenetic relationships of Aspergillus luchuensis ourmia-like virus 1 (AlOLV1). ML tree based on the RdRP alignment of magouliviruses (family *Botourmiaviridae,* order *Ourlivirales*). The LG+I+G4 substitution model was used. Two botouliviruses (family *Botourmiaviridae*) were used as outgroups.
